# Emotion Regulating Attentional Control Abnormalities In Major Depressive Disorder: An Event-Related Potential Study

**DOI:** 10.1038/s41598-017-13626-3

**Published:** 2017-10-19

**Authors:** Bin Hu, Juan Rao, Xiaowei Li, Tong Cao, Jianxiu Li, Dennis Majoe, Jürg Gutknecht

**Affiliations:** 1 School of Information Science & Engineering, Lanzhou University, 730000 Gansu, China; 2 Laboratory for Software Technology-ETH Zurich, Zurich, Switzerland; 3 Institute for Computer Systems-ETH Zurich, Zurich, Switzerland

## Abstract

Major depressive disorders (MDD) exhibit cognitive dysfunction with respect to attention. The deficiencies in cognitive control of emotional information are associated with MDD as compared to healthy controls (HC). However, the brain mechanism underlying emotion that influences the attentional control in MDD necessitates further research. The present study explores the emotion-regulated cognitive competence in MDD at a dynamic attentional stage. Event-Related Potentials (ERPs) were recorded from 35 clinical MDD outpatients and matched HCs by applying a modified affective priming dot-probe paradigm, which consisted of various emotional facial expression pairs. From a dynamic perspective, ERPs combined with sLORETA results showed significant differences among the groups. In compared to HC, 100 ms MDD group exhibited a greater interior-prefrontal N100, sensitive to negative-neutral faces. 200 ms MDD showed an activated parietal-occipital P200 linked to sad face, suggesting that the attentional control ability concentrated on sad mood-congruent cognition. 300 ms, a distinct P300 was observed at dorsolateral parietal cortex, representing a sustained attentional control. Our findings suggested that a negatively sad emotion influenced cognitive attentional control in MDD in the early and late attentional stages of cognition. P200 and P300 might be predictors of potential neurocognitive mechanism underlying the dysregulated attentional control of MDD.

## Introduction

Patients with major depressive disorder (MDD) are associated to reduced cognitive control and regulation of abnormal emotion in high cognitive functions^1,2^. The cognitive control refers to a top-down modulated function of the human brain in attaining focus in order to enhance the processing of task-relevant stimuli and suppress that of the irrelevant stimuli^3^. As a vital aspect of higher brain function, this cognitive ability is not only a type of emotion regulative strategy but also fundamental to human social adaptation^4^. Previous studies suggested that deficits in cognitive control of emotional information are associated with depression^5,6^. Functional magnetic resonance imaging (fMRI) results have elaborated that altered functional brain regions that mediate cognitive control to emotional information might lead to mild-to-moderate symptoms of depression^7^. Recent studies in neural cognition demonstrated that the cognitive processing biased to negative emotion could sustain a depression episode^6,8^. This negative processing bias forms a causal core in the development of the symptoms of depression^9^. Scientists have reported that a negative cognitive process is a crucial factor in determining whether the initially depressed individuals continue a different depressed state, such as mild or severe, transient and persistent^10^. The study also found that cognitive abnormalities in depression varied with mood disturbances^11^. Similarly, Koster *et al*. demonstrated that the bias towards mood-congruent information processing will be viewed as a cognitive vulnerability factor that might influence the cognitive processing^12,13^. Therefore, MDD patients exhibited depression-associated deficits with respect to the control of attention towards negative information^5^. Such deficiencies accompanied by aberrant neural processes may be related to abnormal emotional regulation in MDD^2,5,14^.

Bias and deficits in cognitive control may interfere with emotional regulation and mood state^5^. These deficits are correlated with difficulties in the regulation of emotions^1,15^. According to cognitive theories of depression, the emotion regulation strategy is affected by cognitive control in MDD^16^. The emotion regulation is defined as strategic and automatic processes that influence the emotional response^16,17^. Researchers have reported that the maladaptive emotion regulation strategy is related to several psychological disorders, such as Schizophrenia (SZ)^7^, generalized anxiety disorder (GAD)^18^, and major depressive disorders (MDD), in patients who require the regulation of the abnormal emotion rather frequently than the normal subjects^16^. Another study inferred that emotion regulation dysfunction may result in state and trait negative emotion^15^. A putative mechanism underlying these impairments in MDD is negative mental representation^5^. Previous studies demonstrated that losing anhedonia for a prolonged period, sustained the experience of a negative effect, and lost the experience of positive effect that largely resulted in depressive individuals in inner negative cognition schema^15^. This phenomenon will potentially evoke individual internal responses to reappraise and interpret the social external surroundings in a negative approach (negative cognitive triad)^19^ based on Beck’s cognitive theory of depression^20^. Interestingly, the depressive individuals' internal default nervous system is prone to negative emotional process^16,21^. The negative biased processing also contributes towards maintaining the negative effect and thoughts^19^, which in turn, diminish the reactivity to change the insensitivity of the emotion context (ECI)^12,22^. This latent negative cognitive schema will be activated by the negative-related stimuli according to Beck’s influential cognitive model of depression^15^. Similar results with previous empirical findings indicated that depression is associated with an easy identification of mood-congruent material or rapid orientation towards negative stimuli^16^. Teasdale *et al*. demonstrated that negative bias schema results in MDD in depression-maintaining and cognition-affecting vicious cycle, which led to mood dysfunction^10^. Furthermore, some studies recently provided a similar evidence that this system is reversed by effective habitual filtering processes to modulate the emotional response to the world^23^. The study also defined such involuntary bottom-up filtering for salient stimuli as one of the four basic components of attention^24^. Therefore, the sustained negative mental representations might lead to the dysfunction of internal emotional regulation^16,19^. Moreover, this schema will occupy the maximal amount of the limited attention resources, thereby biasing the attention bias towards negative stimuli.

Attentional control impairments in negative emotion constitute the primary symptoms in patients with MDD. Several pieces of evidence show that the attention bias to negative mood-congruent materials exist in depression, depression co-occurring with anxiety, and other emotional disorders^13,25–27^. A study has also speculated that the negative biased attention could modify the mood and cognition in depression^28^. Other groups also established that biased attention might be the cause of retaining the symptoms of depression. Negative mental representations generate negative effectuated biased attention, which leads to MDD, thereby directing the attention to negative as relevant information and ignoring the positive as irrelevant^29^. The negative mood-congruent attention bias in depression is consistent with the emotional reaction, which is termed as attention facilitation to mood-congruent information^15,30^. On the other hand, attentional control has been shows as a potentially biased process in MDD^6^. Patients MDD have difficulties in both switching their attention from negative-related information to positive and updating the affective response effect. The ability to control the attention switching and maintaining the negative affective response effect is known as attention control (AC)^6^; it can affect the ability to disengage the attention from negative emotional information^16^. Given such difficulties in disengaging from negative stimuli in MDD, the controlling abilities of attentional deployment, as a regulatory strategy, might be impaired^5^. Some studies have postulated a new impaired disengagement model, which elaborates that sustained self-critical negative rumination might produce attention disengagement from negative thoughts^19^. This attentional-disengagement difficulty is a characteristic feature of MDD and at-risk (sub-clinically or remitted depressed) clinically depressed individuals^29^. Recent studies exhibited depression-associated deficits in the control of attention rather than limited processing capacity^5^. Further studies indicated that high-valence negative emotion would elicit attention rather rapidly before the low-valence^31^. Analogously, it is also indicated that emotional-provocative, especially those fear-relevant pictures, can grab the attention effectively^32^. Gotlib *et al*. utilized a dot-probe task with emotional faces as stimuli and found a 1000 ms sustained attentional bias for negative faces in clinically diagnosed depression participants^5^. However, the latest studies^33^ concluded that attention shifting is not only attributed to stimulus-driven bottom-up attention capture but also goal-driven top-down attentional control^34^. Therefore, negative mental representations might be related to impaired attentional control in MDD, thereby giving rise to dysfunctional emotional processing.

## Methods

All methods in this study were performed in accordance with the relevant guidelines, which were declared and approved by the Ethics Committee of the Second Affiliated Hospital of Lanzhou University.

### Participants

Each participant signed an informed consent approved by the Ethics Committee of the Second Affiliated Hospital of Lanzhou University. According to DSM-IV inclusion criteria (American Psychiatry Association 1994), 35 outpatients (15 males and 20 females; 18–55-year-old) diagnosed with depression, as well as 35 healthy controls matched in sex, age, and educational qualifications were recruited for the study. Before the experimental task, the subjects underwent a Mini-International Neuropsychiatric Interview (MINI) and Patient Health Questionnaire-9 (PHQ-9) as an informal clinical interview with the psychiatrist. Those who completed PHQ-9 questionnaire with a score >5 fulfilled the inclusion criteria and were diagnosed as MDD patients. Others comprised the exclusion criteria. Each participant was in good health or corrected-to-normal vision. None of them presented any history of mental disorders, psychotic disease, clinical anxiety, bipolar disorder, or substance abuse or administered any psychotropic medication for a minimum of 4 weeks. Bonus compensation was given to them for participating in this study.

### Stimuli

Stimuli were composed of 60 emotional neutral faces pairs, selected from the standardized native Chinese Facial Affective Picture System (CFAPS)^35^ varied in different emotional valences (positive, negative, and neutral). The emotional facial pictures were selected and classified into four sets as fear, sad, happy, and neutral emotion according to their valence. Two different valences of facial pictures (one belonging to emotional sets, the other as neutral set) were chosen arbitrarily. The number of male and female pictures was equal and resembled each other with respect to physical parameters such as grade, brightness, and size. Subsequently, the pairs of stimuli consisting of neutral and emotional picture, one each, were juxtaposed on the screen for the stimuli pairing. Thus, we obtained emotional-face pair stimuli consisting of 60 emotional faces, including 20 fear-neutral, 20 sad-neutral, and 20 happy-neural faces. These face pairs acted as different valences and could be presented concurrently as random cues in the center of the screen in each trial. The distance between the two pictures was 12 cm with a constant viewing angle of 14.25°. The emotional-stimuli pairs were presented at a uniform black background on a screen with 72 pixels/inch resolution. The whole experiment was conducted in a soundproof and dim lighting condition.

### Procedure

Participants were seated in front of the monitor (17" monitor, 1280 × 1024 resolution, and 60 Hz refresh rate) at a distance of 60 cm and performed an experimental task based on a modified dot-probe paradigm^36^. All relevant instructions were shown on the computer screen initially. Before the experiment begun, the participants were instructed to complete the practical trials (10 trials) to gain familiarity with the task. For the formal experiment, the participants were instructed to concentrate their attention on the compounded emotional face pairs with eyes viewing freely. Consecutively, to ensure a precise response on the location of the dot, they were asked to press the button on the reaction box as quickly and accurately as possible when the dot appeared. The participants must press down the button without any bodily movements including heads or legs, as well as any unnecessary eye movements, saccades, and blinks. They were allowed to rest after finishing each block.

The whole experimental program was designed by E-prime v1.2 (Psychology Software Tools, Inc., Pittsburgh, PA, USA). The session comprised of three blocks (Fear-Neutral, Sad-Neutral, and Happy-Neutral), and each block had 160 trials. Each trial began with a fixed white cross appearing in the central screen at 300 ms and lasted for 300 ms from the beginning. Then, the cross presented centrally on the screen throughout the experiment. A pair of emotional-face stimuli with different valence was presented as a cue and lasted for 500 ms on the screen; one pair individually in a pseudo random order. After a short interval from 100–300 ms, the dot probe was presented randomly for 150 ms as a target in either left or right position of the fixed cross. Concurrently, the participant was asked to identify the spatial location of the ‘dot’ and register his response with the forefinger pressing the button ‘1’ or ‘4’ on the reaction box as quickly as possible. If the dot appeared in the left spatial location of the fixation cross, the subject should press ‘1’; if the dot occurred in the right spatial position, he should press ‘4’ on the response box. An automatic interval of 2000 ms was programmed to receive the participants’ response, or else, the participant would be led into the subsequent trial that was followed by a black screen presented for 600 ms. The procedure continued gradually until a block was finished. Each block was also run in a cycle manner until the whole session was completed. The overall experimental task was accomplished in approximately 25 min.

**Figure 1:**
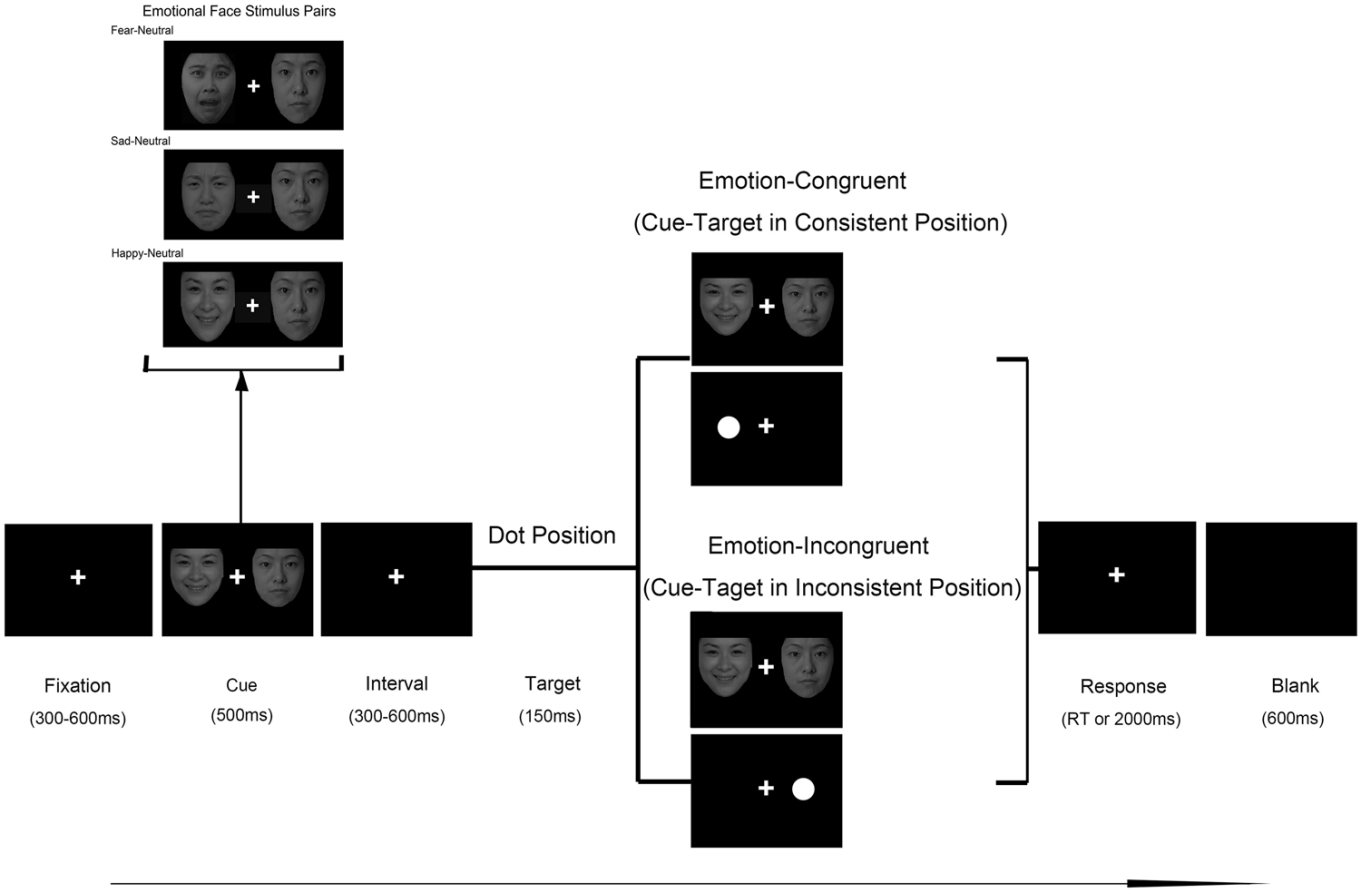
Description of a sample trial sequence. The paradigm used in this study was a modified Dot-Probe Paradigm which included an emotional-neutral face pair (Happy-Neutral, Sad-Neutral, Fear-Neutral) as a cue stimuli and the dot as a target. Each trial began with a fixed white cross appearing in the central screen at 300 ms and lasted for 300 ms from the beginning. Then, the cross presented centrally on the screen throughout the experiment. A pair of emotional-face stimuli with different valence was presented as a cue and lasted for 500 ms on the screen; one pair individually in a pseudo random order. After a short interval from 100–300 ms, the dot probe was presented randomly for 150 ms as a target in either left or right position of the fixed cross. Concurrently, the participant was asked to identify the spatial location of the ‘dot’ and register his response with the forefinger pressing the button ‘1’ or ‘4’ on the reaction box as quickly as possible. If the dot appeared in the left spatial location of the fixation cross, the subject should press ‘1’; if the dot occurred in the right spatial position, he should press ‘4’ on the response box. An automatic interval of 2000 ms was programmed to receive the participants’ response, or else, the participant would be led into the subsequent trial that was followed by a black screen presented for 600 ms. The procedure continued gradually until a block was finished. Each block was also run in a cycle manner until the whole session was completed. The experimental session had three blocks (Happy-Neutral, Sad-Neutral and Fear-Neutral) according to three types of emotional stimulus. Each block had 160 trails. Total trail number was 480.

The schematic experimental procedure of the modified Dot-Probe Paradigm is illustrated in Fig. 1.

### ERP recording and analysis

Continuous ERP brain signal is acquired from a 128-Ag/AgCl electrodes mounted elastic cap (Geodesic Sensor Net, Electrical Geodesics Inc., Oregon Eugene, USA) based on the International 10/20 Standard System. Simultaneously, the behavioral data were recorded from a connected reaction box (Tucker, 1993). The impedance of the electrode was maintained below the recommended value 50 kΩ. The raw data were sampled continuously at 250 Hz and filtered with a band-pass from 0.3–30 Hz off-line. All electrodes were referenced on-line to the CZ position and re-referenced off-line to average off the left and right mastoids electrodes. The electrooculogram (EOG), in each single trial, was eliminated by the principal component analysis (PCA) algorithm. Trials with vertical EOG (VEOG) and horizontal EOG (HEOG) were considered as artifacts and subtracted manually if they occurred simultaneously with our emotional facial-pair presentation. Other trials with reaction times (RT) <100 ms or >2000 ms were excluded from the analysis. The remaining trials were included for further analysis. Using the preprocessing methods consistent with prior ERP studies, we extracted the ERP trials that were time-locked according to both the onset of emotional faces (cue) and that of the dot (probe) in order to evaluate the impact of different emotions on ERPs related to attentional control. After segmenting the ERP data into 800 ms intervals beginning from 200 ms pre-stimulus onset to 600 ms post-stimulus onset, we obtained ERP epochs for each condition to be averaged.

Every epoch elicited by the emotional face cue (Emotional-Neutral facial expression pairs) and target (dot position) was mainly analyzed by an off-line analysis using MATLAB 2013a (The Mathworks, Natick, USA) as well as its toolbox EEGLAB (version 11.2) and ERPLAB toolbox (plugins of EEGLAB, http://www.erpinfo.org/erplab). Based on different emotional cues (fear, sad, and happy face pairs), we analyzed the ERP component involved in early attention control. According to the target (dot position) which appeared in the homolateral or contralateral location of the emotional faces cue, our analysis on late cognitive ERP component was divided into two conditions: emotion-congruent condition (homolateral location: cue-target in consistent position) and emotion-incongruent condition (contralateral location: cue-target in inconsistent position) (Fig. 1).

**Figure 2:**
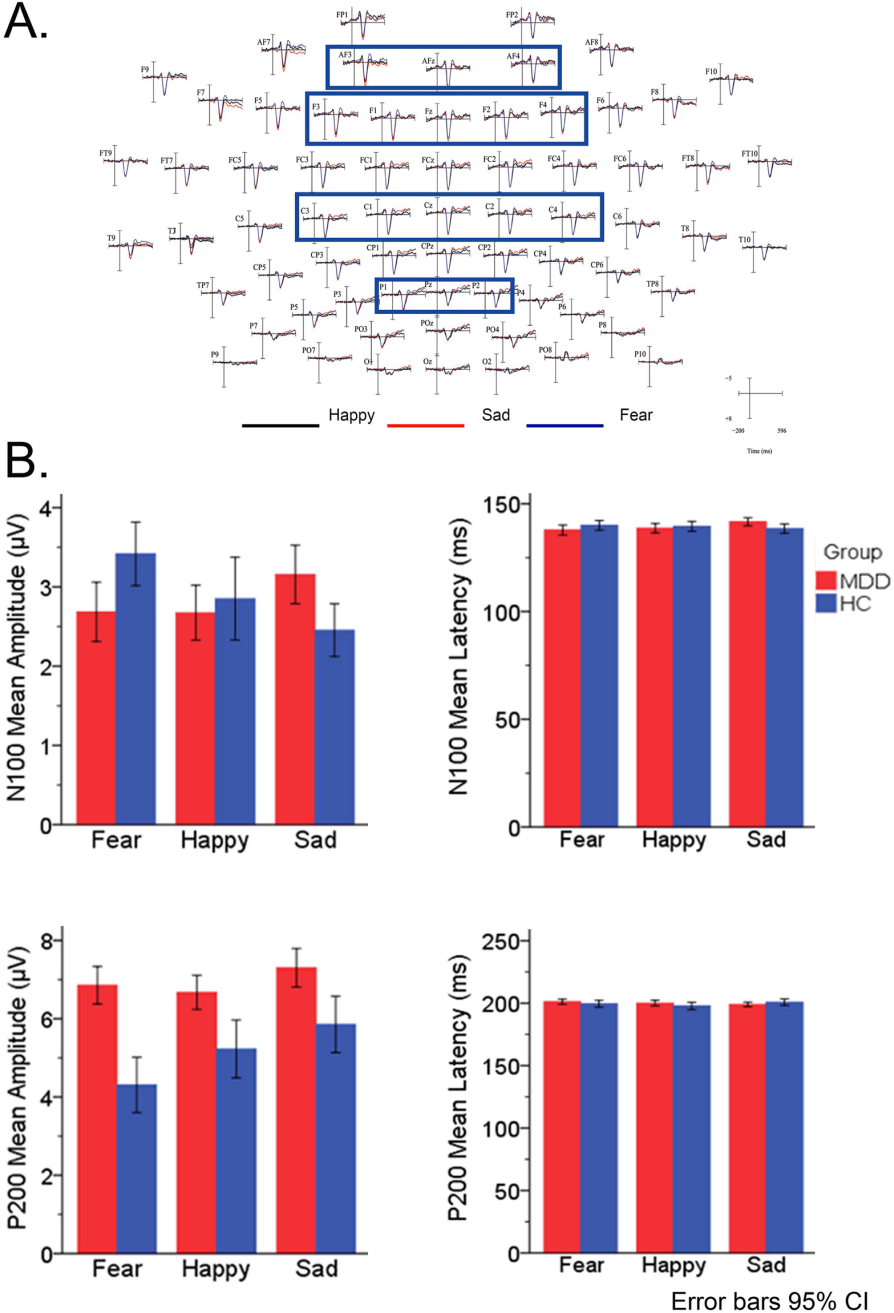
Electrodes, Amplitude and Latency. (**A**) Electrodes related to recording N100 and P200 ERP components. (**B**) Amplitude and Latency of N100 and P200 between MDD and HC in three Emotional-Neutral facial compound stimuli (Sad-Neutral, Fear-Neutral and Happy-Neutral). MDD: Major Depressive Disorder; HC: Healthy Control.

**Figure 3:**
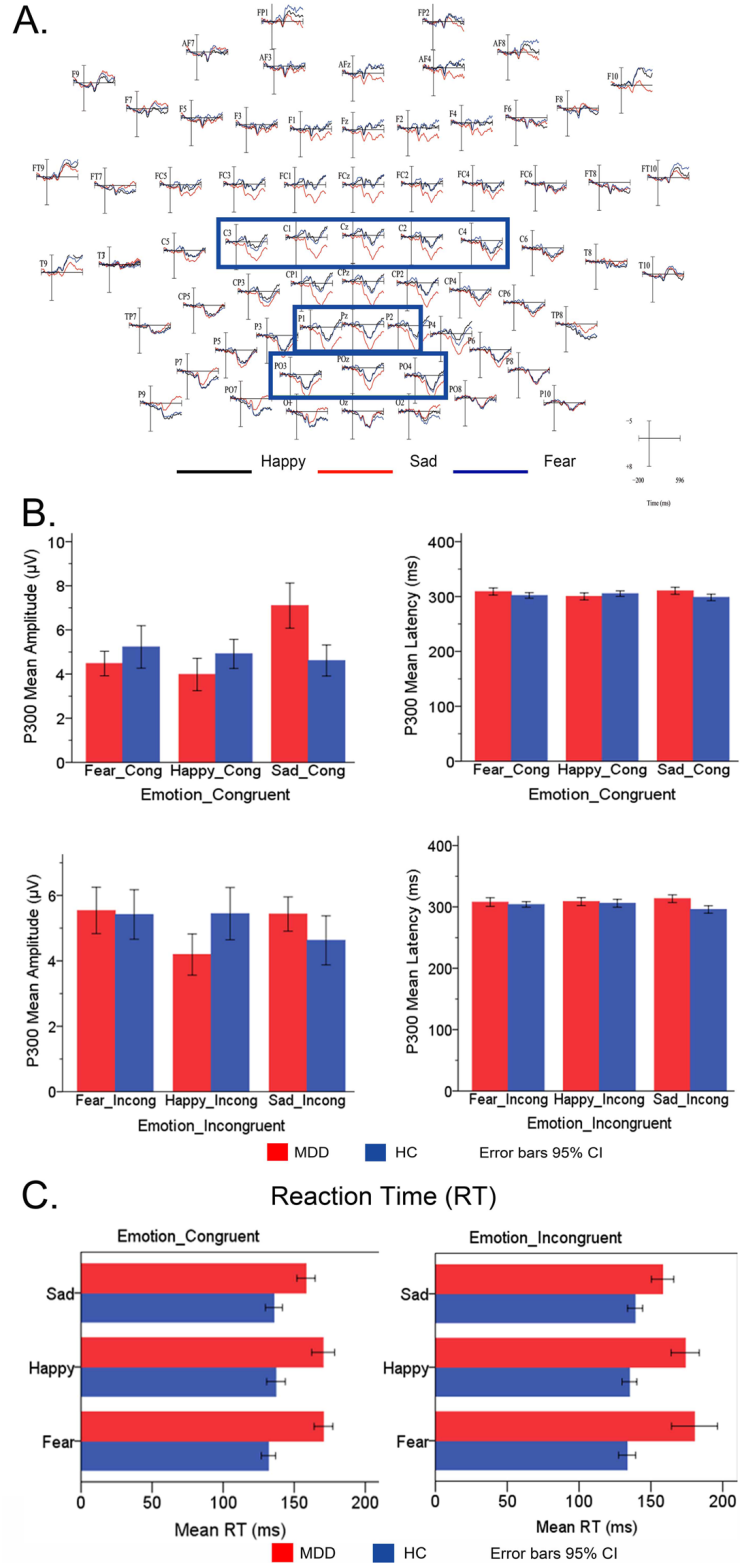
Electrodes, Amplitude and Latency. (**A**) Electrodes related to recording P300 ERP components. (**B**) Amplitude and Latency of P300 between MDD and HC in three Emotional-Neutral facial compound stimuli (Fear-Neutral, Sad-Neutral and Happy-Neutral). (**C**) Group difference in mean Reaction Time (RTs) between MDD and HC under Emotion-Congruent and Emotion-Incongruent conditions. MDD: Major Depressive Disorder; HC: Healthy Control.

The amplitude and latency were calculated using a semi-automatic peak-picking program with ERPLAB tool for statistical analyses. The time-windows locked in each peak were selected. Three ERP components, N100, P200, P300, were found in variant stages of attention processing. According to the computed scalp topographical distribution of grand-averaged ERP activity in the present and previous studies^37^, a set of available electrodes were chosen for statistical analyses of these three components. The following 16 electrode sites (F1, F2, F3, F4, Fz, AF3, AF4, AFz, C1, C2, C3, C4, Cz, P1, P2, and Pz) were selected and classified into four regions: prefrontal (AF3, AFz, AF4), frontal (F1, F2, Fz, F3, F4), central (C1, C2, Cz, C3, C4), and parietal (P1, Pz, P2) (Fig. 2A.) where N100 component (120–140 ms after stimuli pairs onset) had maximal amplitude for statistical analysis. The same set of sites was used for the statistical analysis of P200 component (190–220 ms following the stimulus). To examine the group differences between MDD and HC in three valence emotions on N100, P200 amplitude and latency, a 2 Group (MDD, HC) × 3 Emotional Faces Cue (Happy, Sad, Fear) × 4 ROI (Prefrontal, Frontal, Central, Parietal) design of ANOVA statistical analysis was employed for early attentional stage. The following 11 electrode sites (CP1, CP2, CPz, P1, P3, Pz, PO3, PO4, POz, P2, and P4) were selected and classified into three regions: central (CP1, CPz, CP2), parietal (P1, P3, Pz, P2, P4), and occipital (PO3, PO4, POz) (Fig. 3A) for statistical analysis of P300 component (300–350 ms after stimulus onset). A 2 Group (MDD, HC) × 2 Dot Position (Emotion-Congruent, Emotion-Incongruent) × 3 ROI (Parietal, Occipital, Central) design of ANOVA statistical analysis was conducted for later attentional stage. We selected LSD as an adequate post-hoc test to equalize the assumed variances. Statistical analyses were performed using the SPSS 22 (software version 22, IBM Corp., Chicago, USA).

### Behavioral data recording and analysis

E-Prime software (Psychology Software Tools Inc.) was applied to program the whole experimental procedure and record the accurate response of behavioral reaction times (RT) together with ERP data. The trials were rejected if RT was <100 ms or >2000 ms, and also those trials in which the participants failed to respond were excluded from the analyses. The ANOVA analysis of 2 Group (MDD, HC) × 3 Emotional Faces Cue (Sad, Happy, Fear) × 2 Dot Position (Emotion-Congruent, Emotion-Incongruent) design was performed on mean RT in order to assess the primary effects and interactions.

### Source localization and analysis

ERP associated with sLORETA was analyzed based on the scalp-recorded event-related potential distribution. The standardized Low-Resolution Brain Electromagnetic Tomography (sLORETA) software (http://www.uzh.ch/keyinst/sloreta.htm) was used for computing the three-dimensional cortical distribution of each ERP component density. A three-shell spherical head model registered to the neuroanatomical atlas of Talairach and Tournoux in sLORETA was utilized (1988). Furthermore, the statistical analysis^38^ without the normalization of the raw ERP data was performed. A single t-test was employed for computing the average in a specified interval related to different ERP peak latency in depression and normal control group data; the primary focus was determining the significant group differences between MDD and HC. Then, a randomized SnPM analysis elucidated the brain sources related to ERP components in three emotional modules (Fear, Sad, and Happy) independently at 100, 200, and 300 ms intervals in different attentional stages between MDD and HC group. The statistical differences in brain sources between MDD and HC were shown in Tables 1 and 2.

**Table 1:** Main effects and interaction on Reaction Time (RT) between MDD and HC under Emotion-Congruent and Emotion-Incongruent conditions.

Factor	Levels of Factor		Post Hoc Tests (LSD)		Pairwise Comparisons	
			Std. Error	Sig.	Std. Error	Sig.
**Emotion-Congruent**						
Group	MDD	HC	5.042	0.000	2.911	0.000
	HC	MDD	5.042	0.000	2.911	0.000
Emotion	Fear_Cong	Happy_Cong	3.493	0.562	3.565	0.470
		Sad_Cong	3.493	0.096	3.565	0.239
	Happy_Cong	Fear_Cong	3.493	0.562	3.565	0.470
		Sad_Cong	3.493	0.025	3.565	0.058
	Sad_Cong	Fear_Cong	3.493	0.096	3.565	0.239
		Happy_Cong	3.493	0.025	3.565	0.058
**Emotion-Incongruent**						
Group	MDD	HC	7.669	0.012	4.427	0.000
	HC	MDD	7.699	0.012	4.427	0.000
Emotion	Fear_Incong	Happy_Incong	5.312	0.544	5.421	0.655
		Sad_Incong	5.313	0.037	5.422	0.126
	Happy_Incong	Fear_Incong	5.312	0.544	5.421	0.655
		Sad_Incong	5.313	0.140	5.422	0.279
	Sad_Incong	Fear_Incong	5.313	0.037	5.422	0.126
		Happy_Incong	5.313	0.140	5.422	0.279

The mean difference is significant at the 0.05 level. Adjustment for multiple comparisons: Least Significant Difference (equivalent to no adjustments).

**Table 2:** Brain regions which showed significant differences in activation between MDD and HC group at N100 and P200 latency.

Condition	N1				P2			
	Lobe	Anatomical Region	BA	MNI Coordinates	Lobe	Anatomical Region	BA	MNI Coordinates
**Emotion (MDD** < **HC)**								
Fear	Frontal	IFG	45	(40,20,5)	Frontal	PcG	6	(40,−10,45)
			47	(40,22,4)			2	(40,−19,48)
	Temporal	STG	38	(14,40,−37)		MFG	6	(38,0,43)
		MTG	21	(40,2,−35)				
		ITG	20	(40,−4,−41)				
Sad	Occipital	MOG	18	(−15,−90,15)	Parietal	PCG	1	(50,−25,60)
	Paracentral	PcL	5	(−2,−34,57)				
			6	(−2,−24,65)				
	Frontal	MFG	6	(1,−24,56)				
			11	(−35,41,−21)				
	Temporal	STG	38	(−35,19,−33)				
Happy	Limbic	Pc	31	(−15,−60,30)				
	Occipital	Pc	31	(−13,−92,16)	Occipital	Cuneus	19	(−15,−90,25)
		MOG	18	(40,−10,45)			18	(−15,−82,26)

IFG: Inferior Frontal Gyrus; STG: Superior Temporal Gyrus; MTG: Middle Temporal Gyrus; ITG: Inferior Temporal Gyrus; MOG: Middle Occipital Gyrus; PcL: Paracentral Lobule; MFG: Middle Frontal Gyrus; STG: Superior Temporal Gyrus; BA: Brodmann Area; MNI: Montreal Neurological Institute coordinates; Corrected p < 0.05.

## Results

### Behavioral results

Behavioral data was analyzed according to the participants’ accuracy rate and mean RT. The participants demonstrated a high rate of accuracy in three blocks (Happy-Neural, Sad-Neural, and Fear-Neural) of the task, and the results did not show obvious differences in between the MDD and HC groups.

RT was recorded along with ERP data. A design of ANOVA with 2 Group (MDD, HC) × 3 Emotional Face Cue (Sad, Happy, Fear) × 2 Dot Position (Emotion-Congruent, Emotion-Incongruent) was conducted over RT to examine the main effects of group and emotion under Emotion-Congruent condition as well as emotion-incongruent condition (Fig. 3C).

Our dependent variable RT under two conditions is normally distributed for the groups formed by the combination of different valence: emotion as well as group, as assessed by the Shapiro–Wilk test. A homogeneity of variance between groups was observed by Levene's test for equality of error variance. In the condition of Emotion-Congruent, the results showed a significant effect on Emotion-Congruent RT over Group (*F* (2, 4799) = 117.048, *p* < 0.001) and Emotion: Fear-Congruent and Sad-Congruent (*p* = 0.096), Happy-Congruent, and Sad-Congruent (*p* = 0.025). An obvious interaction over Group at *p* < 0.001 was observed on Emotion-Congruent RT; however, the interaction effects analysis did not reach statistical significant between MDD and HC on Emotion-Congruent RT to different emotions. This might indicate us that the MDD patient exhibits an impaired behavioral ability influenced by different valence emotions, especially, the emotion which is congruent with the endogenous mood. In the condition of Emotion-Incongruent, the results showed a statistically significant interaction between MDD and HC Groups at *p* < 0.001 level on Emotion-Incongruent RT. The analysis of the primary effects showed statistically significant differences over Emotion at Fear-Congruent and Sad-Congruent (*p* = 0.037) in mean Emotion-Incongruent RT between Groups (*F* (2, 4779) = 62.352, *p* = 0.012). For each emotion level, the interaction effects showed no differences in mean Emotion-Incongruent RT. This phenomenon might indicate that disengaging from emotional state and updating the emotional response is rather challenging for the MDD patients (Table 1).

RT shows significant differences between MDD and HC under Emotion-Congruent and Emotion-Incongruent conditions (Fig. 3C). With respect to three different emotional faces (Happy, Sad, and Fear), MDD patients exhibit a longer RT than HC under both congruent and incongruent condition. RT in negative Sad-Congruent emotion in MDD is longer than that in HC, which represents that MDD patients have great difficulty in disengaging from the negative emotion. RT in negative Emotion-Incongruent in MDD is longer than HC, which might be attributed to the response inhibition to different emotions.

## ERP Results

### In the early stage of cognitive attention

#### N100

The significant main effects of group (*F* (2, 144) = 5.503, *p* = 0.019), Emotion (*F* (2, 144) = 7.018, *p* = 0.001) and ROI (*F* (2, 144) = 13.858, *p* < 0.001) over amplitude were evaluated by ANOVA. In addition, the primary effects of group and emotion were not exerted over N100 latency, except for ROI (*F* (2, 144) = 11.203, *p* < 0.001) factor. Moreover, no interaction effects were noted over N100 amplitude. Only a Group × ROI interaction effect (*F* (2, 144) = 5.616, *p* = 0.001) was found over N100 latency. The pairwise comparison revealed that N100 amplitude was considerably different between sad and fear emotion (*p* < 0.001), sad and happy emotion (*p* = 0.024). No obvious difference was found in N100 latency. Furthermore, ROI showed interaction effects over N100 amplitude between parietal and central (*p* = 0.052), parietal and frontal (*p* < 0.001), parietal and prefrontal (*p* < 0.001). On the other hand, interaction effects over N100 latency were obvious between parietal and central (*p* < 0.001), parietal and frontal (*p* < 0.001), parietal and prefrontal (*p* < 0.001).

**Figure 4:**
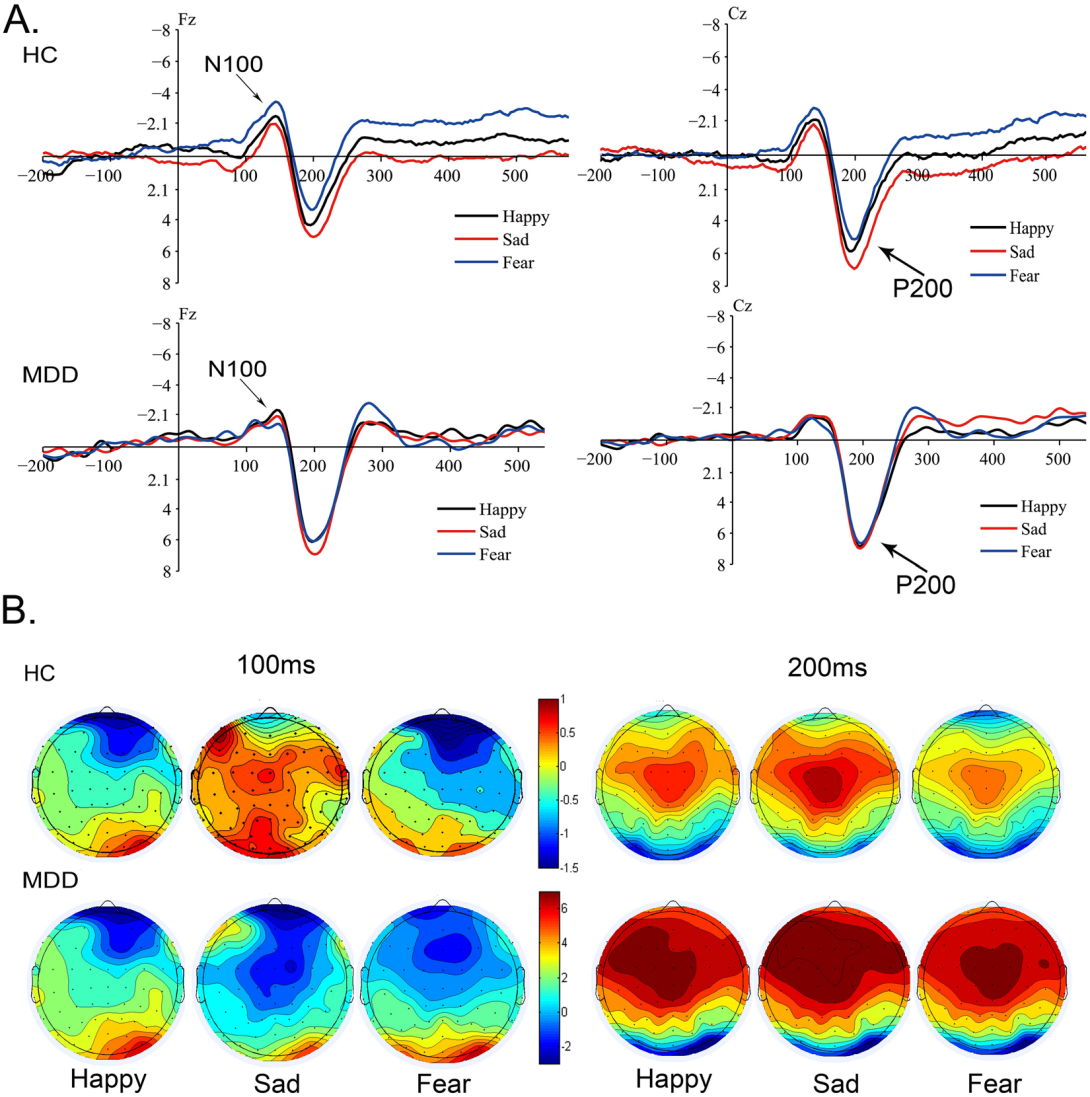
Grand-averaged ERP waveform and Topographic maps of N100 and P200 at 100 ms, 200 ms between MDD and HC. (**A**) Group-averaged ERP waveform relative to N100, P200 components elicited by Emotional-Neutral facial compound stimuli (Sad-Neutral, Fear-Neutral and Happy-Neutral) at Fz, Cz sides between MDD patients and HC at 100 ms and 200 ms. (**B**) Scalp topography of N100 and P200 ERP components generated by Happy-Neutral, Fear-Neutral and Sad-Neutral facial compound stimuli pairs at 100 ms and 200 ms with peak surface potentials (µV) between MDD patients and HC. MDD: Major Depressive Disorder; HC: Healthy Control.

These results imply that fear emotion elicits a rapid alerting of attention in MDD that appeared as lower amplitude and shorter latency, while sad was an intensively potential affect displayed as larger amplitude as well as longer latency, in comparison to the healthy control group (Fig. 4). This phenomenon states that, in the early attention stage, the MDD groups show selective attention, which is prone to negative (Fear, Sad) emotion rather than biases towards catching positive emotion (Happy); especially, MDD patients showed a robust response to negative sad emotion as compared to healthy controls.

#### P200

The significant primary effects of group (*F* (2, 288) = 50.775, *p* < 0.001), emotion (*F* (2, 288) = 5.133, *p* = 0.006), and ROI (*F* (2, 288) = 8.001, *p* < 0.001) were observed over amplitude, reflecting an enhanced P200 in MDD groups than HC controls. Additionally, the P200 latency did not project the main effects of group and emotion, except ROI (*F* (2, 288) = 11.203, *p* < 0.001). No obvious interaction effects of group and emotion over amplitude were found, except for the Group × ROI effect (*F* (2, 288) = 3.442, *p* = 0.016). Other significant interaction effects were not observed over latency. A pairwise comparison revealed that P200 amplitude was obviously different between sad and fear emotion (*p* = 0.002) as well as sad and happy emotion (*p* = 041). No obvious difference was found in P200 latency. The ROIs showed interaction effects over amplitude between parietal and central (*p* = 0.000), parietal and frontal (*p* < 0.001), parietal and prefrontal (*p* = 0.021), while the interaction effect over latency was between parietal and frontal (*p* = 0.004), parietal and prefrontal (*p* = 0.016).

The P200 amplitude in the three emotions of MDD was higher than that in the HC group; the differences in P200 latency under three emotions were not obvious. The P200 amplitude elicited by negative facial expression (fear and sad) was rather higher than that by positive facial emotion (happy) at the early cognitive attentional stage (Fig. 4).

These results suggested that initial attentional control ability was stimulus-driven and affected by external emotional stimulus, especially, the negative sad emotion. P200 reflected the obvious attentional control influenced by emotion, especially at the beginning of cognitive attention. Moreover, this phenomenon might imply that attentional control of depressive patients was sustained in a negative sad mood-congruent rather than fear and happy mood at early attentional recognition.

According to the scalp topographical distribution of grand-averaged ERP N100 and P200 activity, (Fig. 4), MDD patients had almost the same scalp potential voltage value with HC group ranging from −1–0.5 µV at 100 ms; at 200 ms, they showed a higher scalp voltage value from −2–6 µV. In comparison, the healthy participants exhibited a fluctuating value from −1.5–6 µV at 100 and 200 ms. These discrepancies at 100 ms might represent that negative emotion, especially fear and sad, could activate wide brain regions from frontal to central cortex, including dorsal prefrontal and lateral-temporal cortex; whereas at 200 ms, the brain regions related to fear and sad emotion were highly activated in the prefrontal-parietal cortex. Compared to the normal controls, MDD patients showed an increased brain activity to negative emotion.

### In the late stage of cognitive attention

#### P300

A three-way ANOVA was conducted on P300 amplitude and latency under Emotion-Congruent condition and Emotion-Incongruent condition.

Under Emotion-Congruent condition, significant main effects of Emotion-Congruent (*F* (2, 176) = 6.056, *p* = 0.003) and ROI (*F* (2, 176) = 3.148, *p* = 0.044) were observed over P300 amplitude, while the P300 latency showed a significant main effect of ROI (*F* (2, 176) = 4.717, *p* = 0.009). In addition, obvious interaction effects were shown of Group × Emotion-Congruent (*F* (2, 176) = 11.340, *p* < 0.001) over amplitude. A significant interaction effect of Group × ROI (*F* (2, 176) = 4.504, *p* = 0.012) and Group × Emotion-Congruent (*F* (2, 176) = 4.511, *p* = 0.011) over latency was observed. A pairwise comparison indicated that P300 amplitude was obviously different between Sad-Congruent and Fear-Congruent emotion (*p* = 0.017) as well as Sad-Congruent and Happy-Congruent emotion (*p* = 0.001). No obvious difference was found in P300 latency. The ROIs exhibited interaction effects over amplitude between parietal and central (*p* = 0.023), central and occipital (*p* = 0.033), while interaction effects over latency were noted between central and occipital (*p* = 0.033) and parietal and occipital (*p* = 0.037).

**Figure 5:**
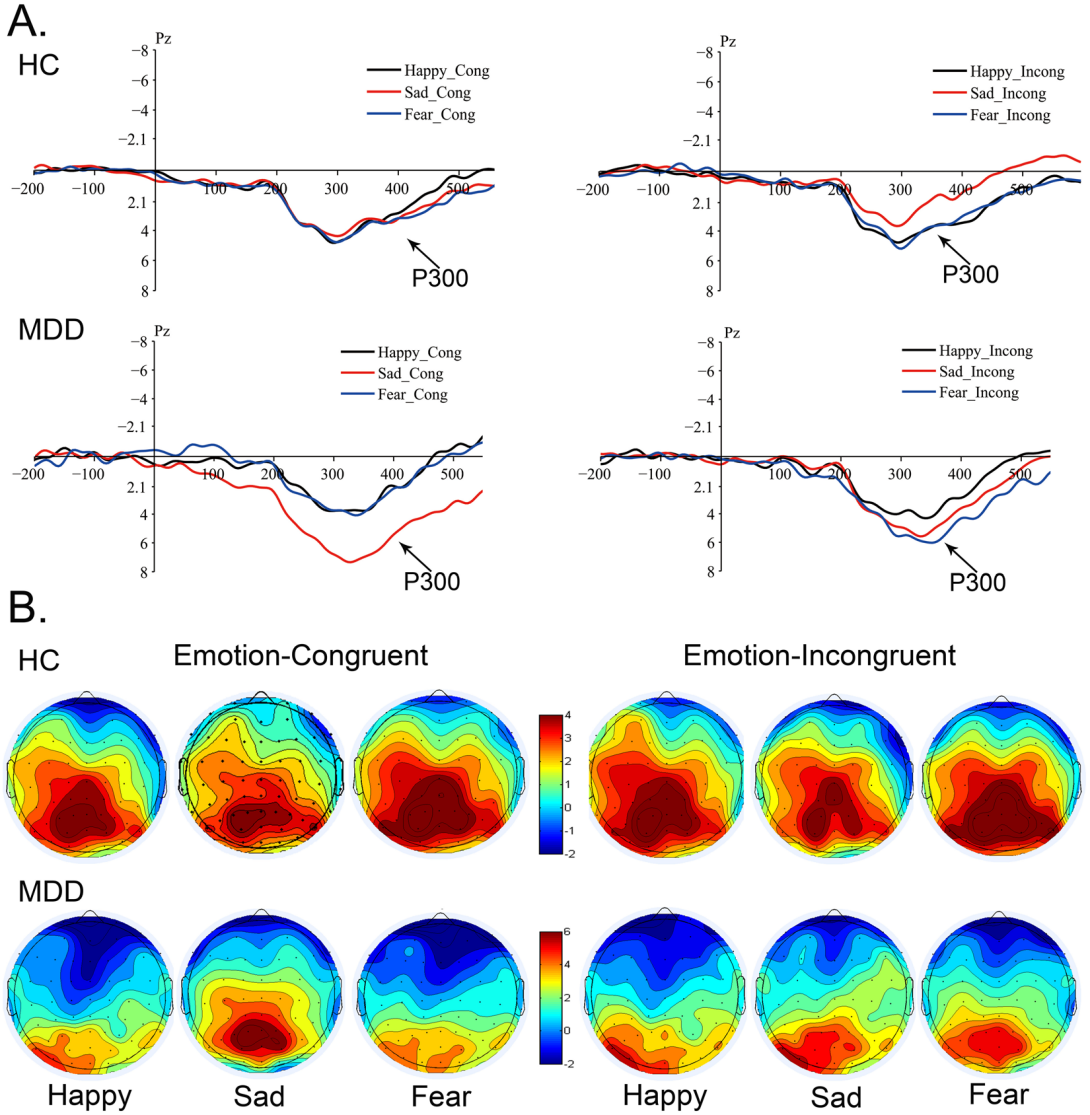
Grand-averaged ERP wave forms and Topographic maps of P300 at 300 ms. (**A**) Grand-averaged ERP waveform of P300 at 300 ms recorded at Cz sides, elicited by Emotion-Neutral facial compound stimuli (Sad-Neutral, Fear-Neutral and Happy-Neutral) under Emotion-Congruent and Emotion-Incongruent conditions between MDD and HC. (**B**) Scalp topography of P300 elicited by Emotion-Neutral facial compound stimuli under Emotion-Congruent and Emotion-Incongruent conditions between MDD and HC. MDD: Major Depressive Disorder; HC: Healthy Control.

Statistical results showed significant difference in Sad-Congruent and Happy-Congruent emotion between MDD patients and HC. As seen in Fig. 5, MDD patients presented a prominently higher amplitude and longer latency of P300 to Sad-Congruent than Fear-Congruent and Happy-Congruent as compared to HC. The enhanced amplitude indicated that attentional psychological resource is supplemented to sustain the attentional control ability in MDD such that it becomes abnormal, thereby resulting in difficulties in disengaging the attention away from negative emotion, especially sad.

These results implied that P300 was engaged in categorizing different valences of emotions in MDD; however, it was not found in HC. Thus, the attentional control was sustained in negatively sad-congruent mood, rather than fear and happy emotion, suggesting that MDD patients focused continuously on sad emotion, which is sustained until the late attentional stage. P300 represented the endurance of attentional control that concentrated on negative emotion, influencing the late cognitive control of attention.

On the contrary, under Emotion-Incongruent condition, no significant main effects of Group, Emotion-Incongruent, and ROI over P300 amplitude were found. P300 latency had significant main effects of Group (*F* (2, 176) = 9.978, *p* = 0.002) and ROI (*F* (2, 176) = 6.040, *p* = 0.003). An obvious interaction effect of a Group × Emotion-Incongruent (*F* (2, 176) = 3.654, *p* = 0.027) over amplitude was observed. Additionally, the interaction effect of a Group × Emotion-Incongruent (*F* (2, 176) = 3.269, *p* = 0.039) was found significantly. A pairwise comparison revealed that P300 amplitude and latency did not differ significantly under Emotion-Incongruent condition. The ROIs showed interaction effects over latency between parietal and central (*p* = 0.009), central and occipital (*p* = 0.001), while no significant interaction effects over amplitude were found.

As shown in Fig. 5, under Emotion-Incongruent condition, the MDD patients exhibited higher amplitude in negative (Sad, Fear)-Incongruent emotion than Happy-Incongruent as compared to the normal controls. The differences in P300 latency between MDD and HC were not obvious, implying that MDD patients did not exhibit attentional control under internal mood-incongruent emotion.

According to the scalp topographical distribution of grand-averaged ERP P300 activity (Fig. 5), the MDD patients presented scalp potential voltage values from −2–6 µV under Emotion-Congruent condition, low scalp potential voltage under Emotion-Incongruent condition. Healthy participants had vibrating scalp potential voltage value from −2–4 µV. The differences might represent that negative emotion, especially sad emotion activated the occipital cortex involved in P300 in MDD patients. The brain regions of HC related to fear and sad emotion were highly activated in parietal-occipital cortex. In comparison, the MDD patients showed a decreased brain activity in these areas related to negative fear and sad emotion both under Emotion-Congruent and Emotion-Incongruent condition. This might reflect that existence of cognitive control abnormality in MDD.

### sLORETA results

**Figure 6:**
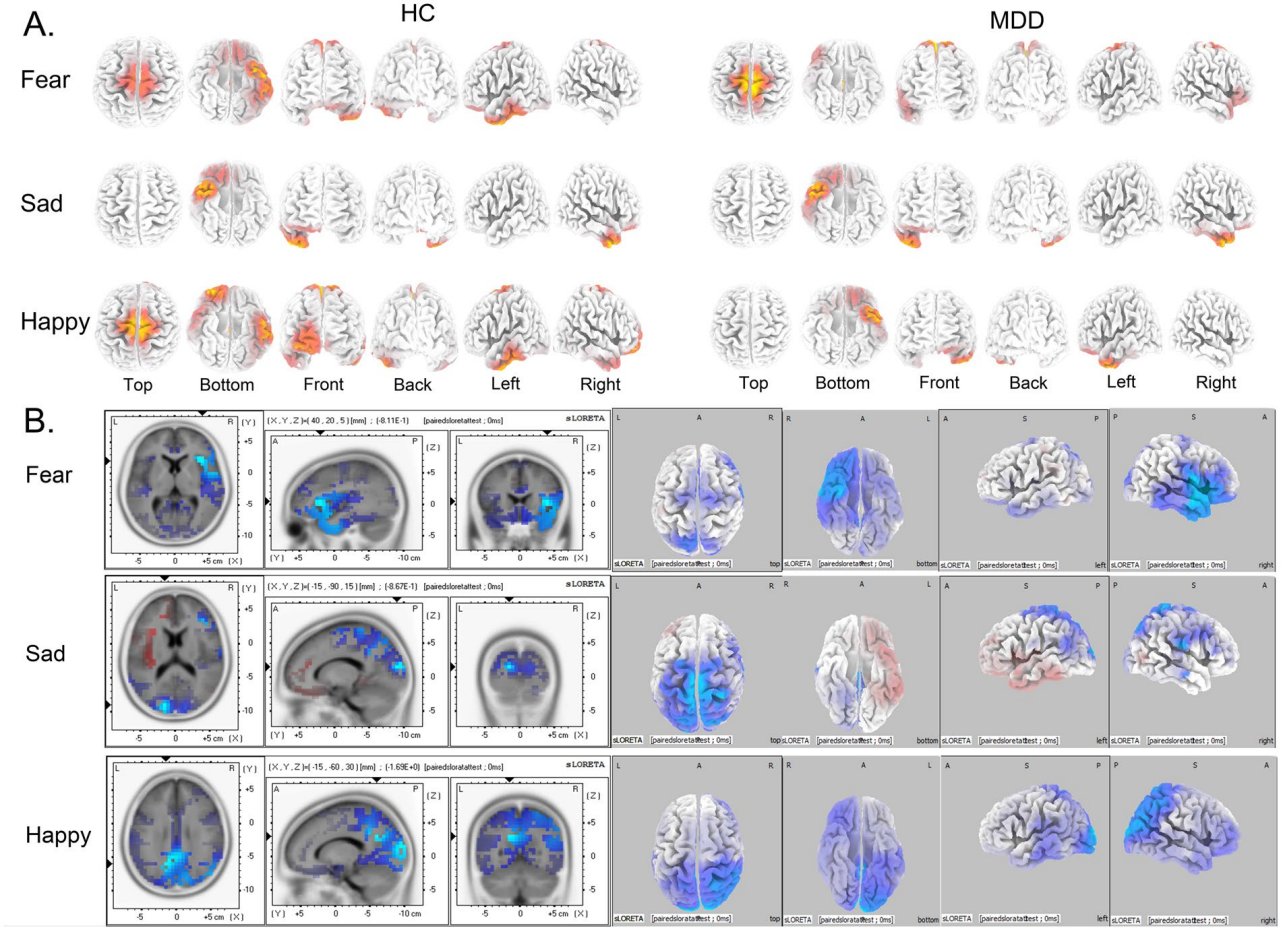
Current density of N100 related brain regions activated by three emotional modules from sLORETA recording at 100 ms. Coordinates in MNI space in mm. Corrected p < 0.05. (**A**) Brain regions activated by three different emotions in MDD and HC groups at 100 ms. (**B**) Differential activation of brain regions at 100 ms between MDD and HC under three emotional facial compound stimuli modules (Fear-Neutral, Sad-Neutral and Happy-Neutral). The areas marked blue represent current density activated in MDD is lower than that in HC (MDD < HC). MDD: Major Depressive Disorder; HC: Healthy Control.

**Figure 7:**
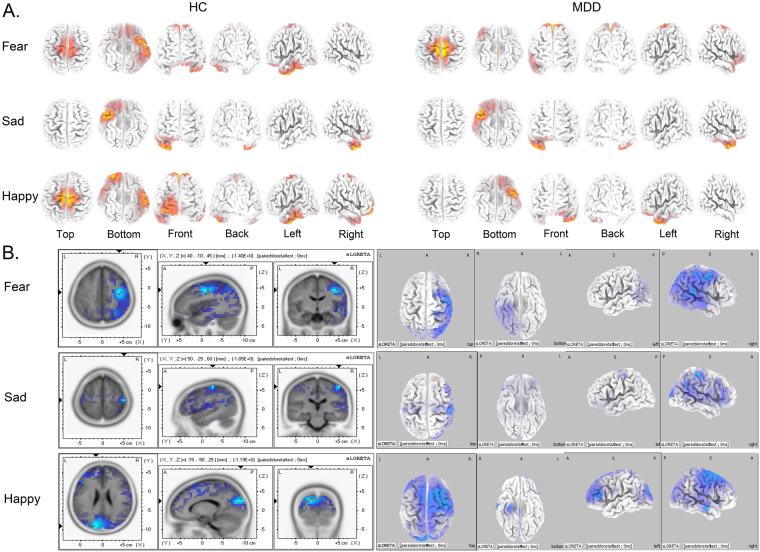
Current density of P200 related brain regions activated by three emotional modules from sLORETA recording at 200 ms. Coordinates in MNI space in mm. Corrected p < 0.05. (**A**) Brain regions activated by three different emotions in MDD and HC groups at 100 ms. (**B**) Differential activation of brain regions at 100 ms between MDD and HC under three emotional facial compound stimuli modules (Fear-Neutral, Sad-Neutral and Happy-Neutral). The areas marked blue represent current density activated in MDD is lower than that in HC (MDD < HC). MDD: Major Depressive Disorder; HC: Healthy Control.

**Figure 8:**
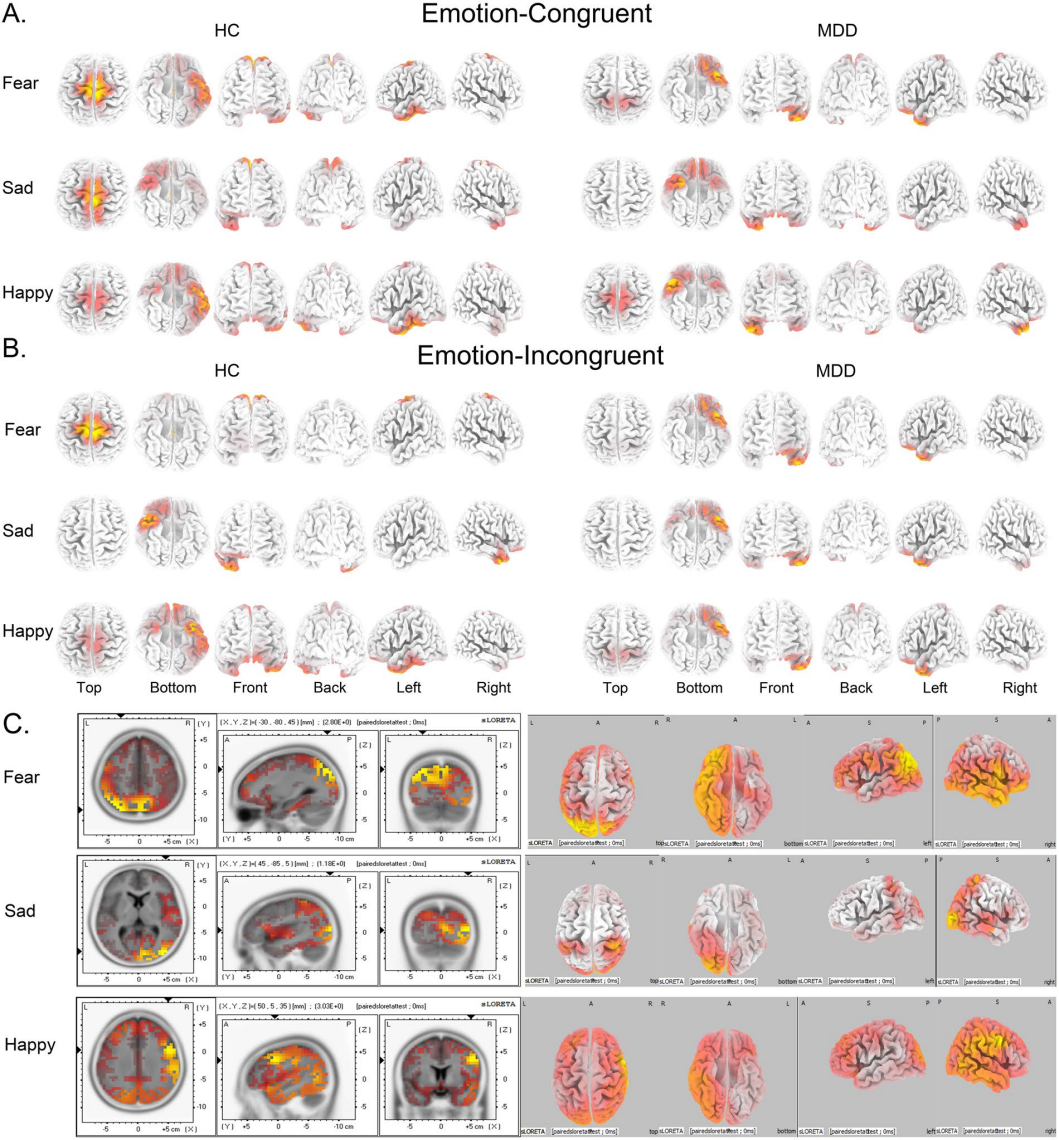
Current density of P300 related brain regions activated by three emotional modules from sLORETA recording at 300 ms. Coordinates in MNI space in mm. Corrected p < 0.05. (**A**) Under Emotion-Congruent condition (Fear-Congruent, Sad-Congruent and Happy-Congruent). (**B**) Under Emotion-Incongruent condition (Fear-Incongruent, Sad-Incongruent and Happy-Incongruent). (**C**) Differential activation of brain regions at 300 ms between MDD and HC under Emotion-Congruent condition.The areas marked red represent current density activated in MDD is higher than that in HC (MDD > HC). MDD: Major Depressive Disorder; HC: Healthy Control.

According to the source location of brain activity related to the three ERP components, we explored the ERP-related cortical regions in spatial and temporal dimensions at dynamic stages of attention. These included inferior prefrontal cortex (IPFC) involved in N100 generations (Fig. 6), parietal-occipital combined with ventrolateral prefrontal cortex (VLPFC) related to P200 (Fig. 7), and the dorsolateral parietal cortex (DLPC) in P300 component (Fig. 8).

### N100 related brain regions

At 100 ms, three different valence emotions activated different brain regions, which were related to attention alerting and attention biases (Fig. 6). With respect to the fear emotion, MDD patients activated saliently in ventral anterior cingulate cortex (vACC, Brodmann 24, 23), primary motor cortex (Brodmann 4, 5), and ventral lateral prefrontal cortex (vlPFC, Brodmann 11). On the other hand, the HC group activated greatly in left ventral temporal gyrus (lvTG, Brodmann 20, 21, 37) and primary motor cortex (Brodmann 4). With respect to the sad emotion, MDD patients activated in a large range of right ventral temporal gyrus (vTG, Brodmann 20) from anterior to posterior, rostral temporal gyrus (rTG, Brodmann 38) and right ventral prefrontal cortex (rvPFC, Brodmann 11); whereas, the HC did not activate similarly. For happy emotion, the MDD patients showed activities in the left ventral temporal gyrus (lvTG, Brodmann 20) and inferior prefrontal cortex (iPFC, Brodmann 11, 47), whereas the HC group activated greatly in the left ventral middle temporal gyrus (vmTG, Brodmann 20, 37) and right prefrontal cortex (rPFC, Brodmann 10, 11). The differential activated brain regions relative to three emotions (Fear, Sad, and Happy) at 100 ms were listed in Table 2 and Fig. 6.

These results showed that different valence emotions activated different brain regions between MDD patients and HC controls at the early stage of attention processing. For the fear emotion, MDD group showed an obvious avoidance of the fear as compared to the HC group, which could be found in both intracerebral sources (Fig. 6) and short N100 latency combined with low amplitude. For the sad emotion, MDD patients exhibited an enhanced scope of activated ventral temporal gyrus as compared to HC, which indicated that MDD patients had increased emotional activities towards sad emotion and decreased responsiveness to happy emotion. For the happy emotion, the MDD group exhibited decreased brain activities as compared to HC controls, which suggested that negative cognitive schema rendered abnormality to the initial attentional control abilities in MDD. As a result, the MDD patients could not shift their attention from sad to happy emotion unlike the HC group.

### P200 related brain regions

At 200 ms, three different valence emotions exhibited different effects on initial attentional control in intracerebral sources related to P200 (Fig. 7). In the case of fear emotion, MDD group activated intensively in the dorsal posterior cingulate cortex (dpCC, Brodmann 31) and primary motor cortex (Brodmann 5), while the HC group activated in the left ventral temporal cortex (vTG, Brodmann 20, 21, 37) and inferior ventral prefrontal cortex (ivPFC, Brodmann 11). With respect to sad emotion, the MDD group showed enhanced activities in the right ventral temporal gyrus (vTG, Brodmann 20) and right rostral temporal gyrus (Brodmann 38) than the HC controls. Regard the happy emotion, the MDD patients showed decreased activities in the left anterior temporal gyrus (laTG, Brodmann 38) and inferior prefrontal cortex (iPFC, Brodmann 11); whereas, the HC group activated greatly in the left ventral middle temporal gyrus (vmTG, Brodmann 20, 37) and right prefrontal cortex (rPFC, Brodmann 10,11). The differentially activated brain regions relative to three emotions (Fear, Sad, and Happy) at 200 ms were listed in Table 2 and Fig. 7.

These results showed that the brain activities were affected by different valence emotions between MDD patients and HC controls at the early stage of attention processing. For fear emotion, the MDD group showed obviously enhanced cognitive ability of emotion processing affecting the recognition as compared to the HC group, and both could be found in large P200 amplitude and activated dorsal posterior cingulate cortex (Fig. 7). For the sad emotion, MDD patients exhibited an enhanced range of activated ventral temporal gyrus (vTG) and inferior prefrontal cortex (iPFC) as compared to HC, which indicated that MDD patients showed an increased cognitive control related to emotion and intensive attentional control to sad emotion, thereby inhibiting the control ability of shifting towards the happy emotion. For happy emotion, the MDD patients activated weakly in the left ventral temporal gyrus (lrTG), rostral temporal gyrus (rTG), and right prefrontal cortex (rPFC) than that in the HC group, which suggested that MDD patients exhibited altered attentional control to happy emotion as compared to HC. During this period, attentional control ability was effectuated, which was embodied in a stronger P200 amplitude and shorter P200 latency.

### P300 related brain regions

At 300 ms, three different valence emotions had obviously different effects on sustained attentional control in intracerebral sources related to P300 under Emotion-Congruent and Emotion-Incongruent conditions (Fig. 8). For Fear-Congruent condition, the MDD patients were found to be activated in the left rostral temporal gyrus (lrTG, Brodmann 38), inferior ventral prefrontal cortex (ivPFC, Brodmann 11, 45), and primary motor cortex (Brodmann 6, 7). On the other hand, the HC group was activated in the left ventral temporal cortex (lvTG, Brodmann 20, 21, 37), dorsal posterior cingulate cortex (dpCC, Brodmann 31), ventral cingulate cortex (vCC, Brodmann 24), and primary motor cortex (Brodmann 4, 5). With respect to the Sad-Congruent condition, the MDD group showed obvious activation in the bilateral inferior prefrontal cortex (iPFC, Brodmann 11, 47), right rostral temporal gyrus (rTG, Brodmann 38), and left rostral temporal gyrus, while the HC group was activated in the right temporal gyrus (rTG, Brodmann 20), primary motor cortex (Brodmann 6, 7), and lateral right inferior prefrontal cortex (rIPFC, Brodmann 11). For Happy-Congruent emotional condition, the MDD patients were activated in the lateral left rostral temporal gyrus (lrTG, Brodmann 38) and lateral inferior prefrontal cortex (iPFC, Brodmann 11, 47), whereas the HC group was activated in the left temporal gyrus (lTG, Brodmann 20, 37), right inferior middle prefrontal cortex (imPFC, Brodmann 10), and primary motor cortex (Brodmann 6, 7). The differentially activated brain regions relative to Emotion-Congruent at 300 ms were listed in Table 3 and Fig. 8.

**Table 3:** Brain regions which showed significant differences in activation between MDD and HC group at P300 latency under Emotion-Congruent condition.

Condition	Lobe	Anatomical Region	BA	MNI Coordinates
**Emotion-Congruent (MDD** > **HC)**				
Fear-Con	Parietal	SPL	7	(−30,−80,45)
		Pc	7	(8,−68,39)
		IPL	7	(−41,−68,74)
			40	(−45,−66,37)
	Temporal	STG	39	(−45,−61,29)
	Occipital	STG	19	(−41,−78,23)
Sad-Con	Occipital	MOG	19	(45,−85,5)
		Cuneus	17	(37,−43,54)
	Parietal	IPL	40	(12,−92,7)
Happy-Con	Frontal	IFG	9	(50,5,35)
		MFG	9	(50,6,39)
		PcG	6	(50,1,33)

SPL: Superior Parietal Lobule; Pc: Precuneus; IPL: Inferior Parietal Lobule; STG: Superior Temporal Gyrus; MOG: Middle Occipital Gyrus; IFG: Inferior Frontal Gyrus; MFG: Middle Frontal Gyrus; MTG: Middle Temporal Gyrus; ITG: Inferior Temporal Gyrus;; PcL: Paracentral Lobule; PcG: Postcentral Gyrus; BA: Brodmann Area; MNI: Montreal Neurological Institute coordinates; Corrected p < 0.05.

Under Emotion-Incongruent condition (Fear-Incongruent, Sad-Incongruent, and Happy-Incongruent), the MDD group was activated in the left rostral temporal gyrus (rTG, Brodmann 38) and inferior prefrontal cortex (iPFC, Brodmann 11); whereas the HC group was activated in the dorsal posterior cingulate cortex (dpCC, Brodmann 31) for fear emotion, right rostral temporal gyrus (rTG, Brodmann 38) for sad emotion, and left ventral temporal gyrus (lvTG, Brodmann 20, 21, 37) for happy emotion. Those differentially activated brain regions under Emotion-Incongruent were neither analyzed nor shown in Fig. 8 as it was not closely related to our hypothesis.

These results showed that brain activities were influenced by different Emotion-Congruent conditions between MDD patients and HC controls at a late stage of attention processing. For Fear-Congruent emotion, the MDD group showed weakened cognitive ability of attention processing towards the fear emotion as compared to HC, which could be found both in attenuated P300 amplitude coupled with a prolonged latency and diminished dorsal posterior cingulate cortex (Fig. 8). For Sad-Congruent emotion, the MDD patients exhibited an enhanced activity of bilateral inferior prefrontal cortex and activated right rostral temporal activity as compared to the HC group, which indicated that MDD patients showed continuously increasing emotional cognitive control that appeared as intensive attentional control of sad emotion. For Happy-Congruent emotion, the MDD patients activated weakly in the left ventral temporal gyrus (vTG), strong in the right rostral temporal gyrus (rTG), and right ventral prefrontal cortex (vPFC), while the HC group activated in the left ventral temporal gyrus (vTG) and decreased in the right rostral temporal gyrus (rTG). This phenomenon represented that the MDD patients exhibited aberrantly decreased attentional control towards happy emotion as compared to the HC group. During this period, the attentional control ability was already formed that began to affect the cognitive decision in MDD patients. This decision was embodied in a strong P300 amplitude and prolonged P300 latency, especially towards sad emotion. In addition, the RT among three emotions under Emotion-Congruent condition also proved that the responsiveness of MDD was sensitive-to-sad emotion, fear was designated as second, followed by the happy emotion. This indicated that MDD patients were susceptible to empathizing with their internal sad mood that was effectually sustained without any update.

### Brain Regions of sad-related attentional control in MDD changed dynamically from 200–300 ms

**Figure 9:**
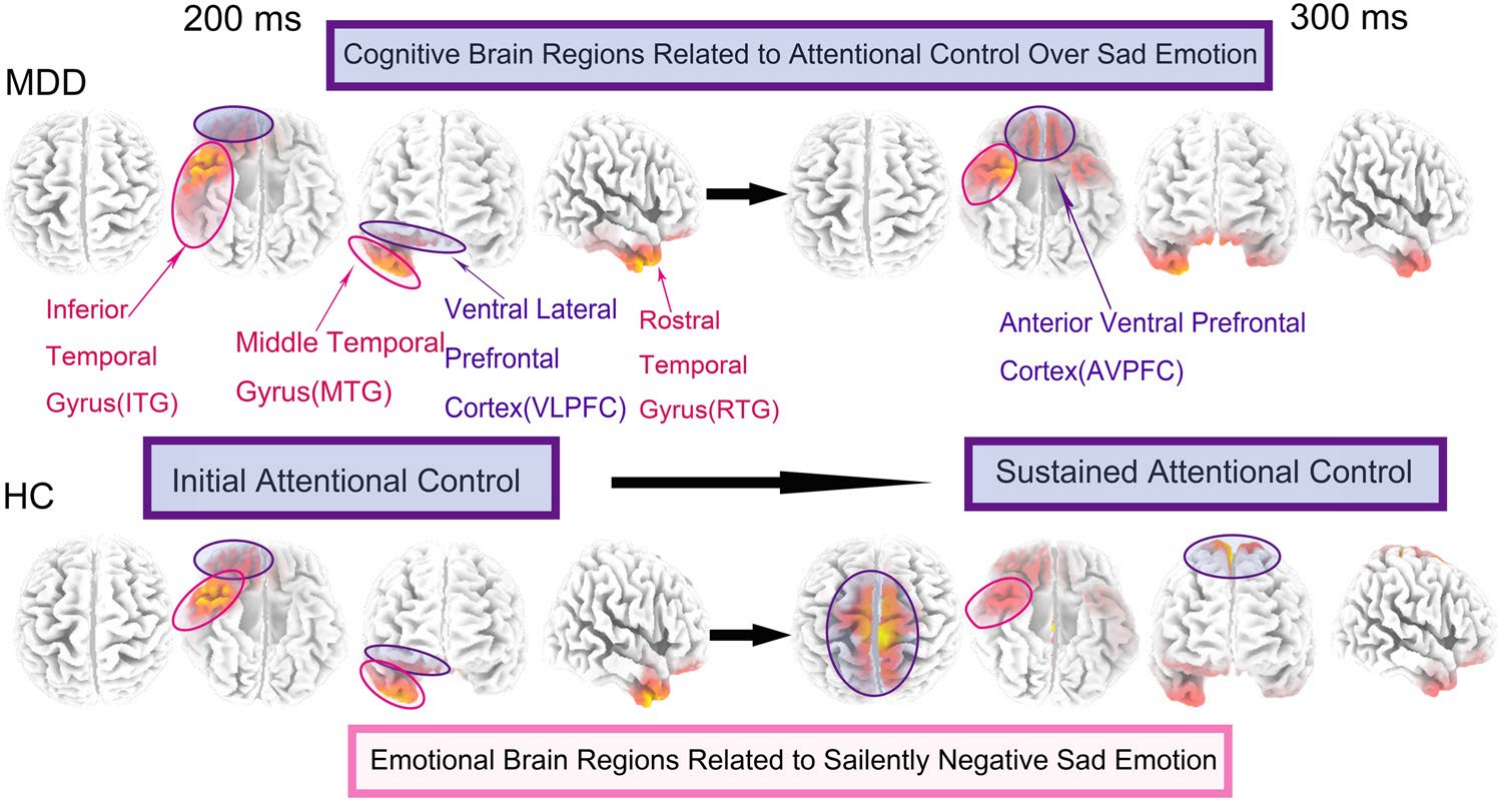
The hypothetical brain circuitry of sad emotion influencing attentional control in facial expression processing in MDD patients. From dynamic perspective which begins from 200 ms (initial attentional control) to 300 ms (sustained attentional control), MDD patients exhibit a sad-related developing procedure, changing from inferior temporal gyrus (ITG) to bilateral ventral prefrontal (AVPFC), which is quite different with HC. MDD patients show abnormal changes in attentional control ability compared with healthy controls in this periods. MDD: Major Depressive Disorder; HC: Healthy Control.

From a consecutively dynamic perspective of 200**–**300 ms, MDD patients exhibited a sad-related brain activity that altered from inferior temporal gyrus (ITG) to rostral temporal gyrus (RTG), activating an intensively larger area as compared to the HC group. In this process, the activated brain regions of HC were involved in the rostral temporal gyrus (RTG, Brodmann 38) as well as dorsolateral parietal cortex (DLPC), which showed normal attentional control ability in the HC group. In the case of MDD patients, increased abnormal attentional control over sad emotion was exhibited involving the bilateral anterior ventral prefrontal (AVPFC) (Fig. 9). These results implied that MDD patients showed abnormal changes in attentional control ability as compared to healthy controls.

## Discussion

The present study unraveled the dynamic brain mechanisms underlying the attentional control in MDD patients, which was affected by different valence emotions. Herein, three aspects were addressed. Firstly, we investigated the hypothesis that attentional control would be affected by salient emotional stimuli in different attentional stages. Secondly, the underlying mechanisms of attentional control in dynamic cognitive course were explored. Finally, the neuropsychological brain circuitry of attentional control related to emotion processing was also elucidated in MDD patients.

### Attentional control affected by salient negative emotion

In this exploratory study, we designed a modified dot-probe attentional task with emotional-faces in order to investigate the attentional cognitive control in MDD. The results led to the hypothesis that emotion can regulate the attentional control in early and late attentional stages in MDD.

In the early and late stages of cognitive processing, attentional control was altered by salient negative emotion. A short N100 latency to the negative fear emotion and high amplitude of P200 over sad emotion in the early stage indicated an enhanced attention alert and circumvention of fear emotion that might be a transient property, while sad emotion was probably affected in MDD. This phenomenon might imply that the attentional control ability of depressive patients was maintained in negative sad mood-congruent rather than fear and happy mood at early attentional processing. P200 serves as a biological marker, which represents cognitive and psychological characteristics, reflecting the initial attentional control ability in MDD influenced by emotion at the beginning of cognitive attention. These features suggested that the initial attentional control ability was stimulus-driven and affected by external emotional stimuli, especially, negative sad emotion. On the other hand, increased P300 amplitude and prolonged latency to sad stimuli in late attentional stage might suggest attentional control affected and modulated by the sad emotion. Thus, it can be inferred that the attentional control was sustained in a negatively sad-congruent mood, rather than fear and happy emotion. Therefore, it can be inferred that MDD patients continually focus on the sad emotion, which is sustained from the beginning until the late attentional stage. P300 represented enduring attentional control ability concentrating on negative emotion, which putatively affected the attentional control in the late attentional stage. Such modulated attentional control abnormalities in MDD differ distinctly from HC.

RT in the late cognitive state can be considered as another symbol that might explain the cognitive attention control ability of MDD. As can be seen in our results, the MDD patients are predisposed towards longer RTs under different Emotion-Congruent conditions than the HC group. Specifically, the RTs of MDD in negative Fear-Congruent emotion and positive Happy-Congruent emotion were larger than that in the Sad-Congruent emotion. Strikingly, short RTs to Sad-Congruent emotion were observed, which might be attributed to the altered mood-state-dependent emotional reactivity produced by MDD^4^. These results might indicate that the internal sad emotion of MDD patients’ was evoked by external sad facial expression and the patients were immersed in such sad-congruent mood, which rendered them susceptible to respond to sad emotion, shown as the short reaction time. This also brought about great difficulties in updating, shifting, and disengaging from negative emotion to positive in MDD, termed as Attention Control (AC). This abnormal control ability led to prolonged RTs in the negative fear emotion of MDD than that in HC groups. The long RT of MDD in positive-congruent emotion as compared to HC might be attributed to the impulsive reactivity stimulated by the ambivalence of positive and negative emotion, with these opposite emotions being both high emotional intensity and correlated. This phenomenon was proved with respect to the Emotion-Incongruent condition, RTs in MDD were longer than that in HC due to the response inhibition to different emotions.

### Attentional control-related brain mechanisms

Unlike previous studies focusing on MDD attention bias to negative emotions, the present study investigated how emotion regulates attentional control in dynamic attentional processing in patients with MDD. The results demonstrated that salient emotional facial expression might affect and modulate the attentional control by eliciting internal mood-congruent affection.

Based on high temporal resolution of ERP recording technology, ERPs provide precise brain activities, which reflect potential attentional cognitive guidelines. Owing to satisfactory features, previous studies suggested that ERP components associated with attention in different periods can be analyzed^39^. The current ERP results also indicated the potential role of attentional control in MDD. We obtained the inferior-frontal cortex N100 in the early state, a cue (emotional facial pairs)-elicited distinct ERP component, which showed a biased insight of MDD into negative stimuli at the early stage of attention. Parietal-occipital lobe related to P200 reflected that the attentional control ability concentrated on the individuals’ internal congruent-mood, which resonates with external emotional stimuli. Moreover, P300 suggested the occurrence of changes in cognitive ability, responsible for maintaining the attentional control on negative emotion in the late attentional stage. Compared to healthy controls, the ERP results inferred that depressive patients showed an increased N100 amplitude and distinctly higher amplitude of P200 in the early cognitive attentional stage, whereas an increased P300 amplitude in the late attention cognitive processing.

On the other hand, the peak latency of N100, P200, and P300 were specific to MDD. N100 latency in MDD elicited by positive emotion (Happy) is slightly longer than that elicited by negative (Fear) emotion. This characteristic probably indicated that the MDD group circumvented the fear emotion rather than perceiving the happy emotion in the early attentional processing stage. At this stage, among the three emotional stimuli, sad emotion elicited the longest N100 latency in MDD as compared to the HC group. With respect to P200 latency, no obvious differences were observed among different emotions; only sad emotion gave rise to a short latency as it resonated with the internal sad-congruent emotion. Regarding P300, negative (fear and sad) emotion elicited a prolonged latency than positive (happy) emotion in MDD. These findings suggested that negative emotion altered the attentional cognitive control in the early and late attentional stage.

### Initial attentional control

#### Distinct P200 represent initial attentional control

The distinct component N100 relative to early attentional state was significantly different at the inferior-middle prefrontal cortex (IMPFC) as compared to health controls. According to previous studies, N100 wave indicated the initial sensory processing mechanisms and early selective attention^30^. Other studies indicated that fearful, disgust, and happy faces elicited a larger N170 amplitude than neutral faces. Fearful facial expression elicited a prominent negative N100 component than happy and neural faces^15^. Some groups also found a greater N100 for threatening cues than the pleasant cues in early attentional process^40^. An early anterior N100 elicited by fearful faces were larger than happy or neutral faces^41^. In the current study, N100 amplitude was larger over sad than fear and happy faces, which was not in agreement with that from previous studies^15^. This phenomenon might be attributed to the biased attention, which is prone to be preoccupied by negative emotional cues in depressed patients. At an antecedent attention stage, the saliently negative emotional stimuli might grab attention more rapidly than the neutral stimuli in MDD, which is consistent with the stimuli-driven cognitive process of ‘hot’ model (emotion-valence)^42^. Together, an early attentional-orienting bias to negative emotion in MDD is observed^25^. In the subsequent cognitive processing, a biased negative affection occurs in late cognitive stage^43^. The current findings indicated that the MDD groups preferentially gave rise to intensively potential sad emotion in contrast to healthy controls in the early attentional stage. On the other hand, the latency of N100 to sad facial stimuli was longer than fear, suggesting that sad emotion exhibited a state-trait. The sad emotional traits in MDD were sustained for a prolonged duration beginning from perception of negative emotion to its succeeding processing, i.e., in the early attentional stage, fear emotion presented a transient-trait feature, while sad emotion reflected state-trait features in MDD.

Distinct P200 represented an initial attentional control. P200 was commonly reported to represent the initial differences from task-relevant and task-irrelevant stimuli. It has been associated with early attention to emotional stimuli^37^. Researchers also established that P200 was a biomarker of attentional impairment in depression disorder patients. In a study focusing on emotional facial expression associated with depression, larger amplitude and longer latency of P200 in the MDD group was observed in intensely sad emotional facial expressions^44^. In another auditory research, P200 proposed a potential mechanism underlying attentional impairment in depressive patients, especially in severe depression cases^45^. Some depressive rTMS (repetitive transcranial magnetic stimulation) treatment studies demonstrated that altered P200 amplitude and latency were related to improvement depressive symptom post-rTMS treatment. This finding implied that P200 was involved in different depressive symptoms^46^. Jessica J. Green also demonstrated that parietal activated P200 predicted top-down attentional control in initial signal processing^47^.

In the current study, attentional control component P200 was significantly different in parietal-central cortex between MDD and HC groups. The P200 amplitude exhibited a larger deflection to negative emotional cues (fear and sad) than happy faces in MDD. In addition, we found that the parietal-occipital P200 was an obvious mark with initial top-down attentional control in cognitive processing. This finding indicated that exogenous emotional stimuli might influence the shift in attention. Therefore,, the attention may be oriented towards negative sad information, which is evoked by exogenous emotional stimuli, and such negative emotion would obligate the reallocation of attention resources, thereby leading to the inhibition of attention shifting from negative to positive emotion. Thus, an MDD patient exhibited impaired attentional control ability to sad emotion as compared to healthy controls.

### Sustained attentional control

#### Prominent P300 represents sustained attentional control

Cognitive component P300 firstly reported by Sutton *et al*. (1965) is regarded as an ERP index to assess the neurocognitive effects. In this study, P300 relative to the late cognitive stage showed a considerable difference in the amplitude between MDD and HC groups, which reflected the response of strong attentional control ability in order to modulate the affective stimuli. Previous studies demonstrated that the amplitude of P300 represents appropriate allocation of psychological resources, such as attention, expectation, and stimulus equality, while P300 latency might reflect the evaluation time of the stimulus^37,48^. Furthermore, P300 was involved with automatic orienting response to new, salient, or rare stimuli for working memory, contextual updating, and response modulation^30^. Other studies demonstrated that emotional stimuli can elicit a high P300 amplitude than neutral stimuli^44^.

Additionally, new findings indicated that parameters of P300 can assess the specific psychosis and cognitive dimension of depression^49^. P300 was speculated as the neurophysiological index of cognitive dysfunction in depression^50^, as well as the prolonged P300 latency as the state marker for MDD^49^. Therefore, further studies on P300 amplitude and latency are needed. In this study, the MDD group showed higher amplitude relative to sad facial emotion rather than that relative to fear and happy facial expression, which represented further attentional resources distributed continuously to sad emotion. Moreover, increased higher P300 amplitude and longer latency might reflect attentional control ability, which was altered by negative sad mood state in MDD, thereby exhibiting that attentional control is affected and altered saliently by negative emotional stimuli.

In conclusion, great discrepancies were observed in attentional cognitive processing between MDD and healthy controls. MDD patients presented remarkable abnormalities in the early and late attentional stage, which indicated that the individual internal negative cognition schema might influence the subsequent steps of cognitive control processing, including perception, attention, appraisal, and decision. This finding was in agreement with previous results^15^. The negative emotional face stimuli resonated the inner negative mental representation, resulting in negative biased attention in the early stage. Thereby, the psychological cognitive schema will effectuate the succeeding cognitive processing. As a result, in the late attentional stage, the attentional control and the type of cognitive control ability would be altered and sustained to negative emotion. Consequently, the regulation of emotional strategies in MDD were abnormal. If this ability was impaired for a prolonged period, patients with MDD would be lost in the dysfunctional cognition and would exhibit abnormal affective symptoms. Previous studies have indicated that altered cognitive control ability in MDD associated with levels of severity in depressive symptoms would be interfered by emotional stimuli^7^. Based on this study of emotional facial priming attention paradigm, the differences in cognitive attentional control between the MDD and HC groups occurred in the early and late cognitive stage. Taken together, the attentional control of the brain mechanism was influenced and regulated by emotion, especially salient sad emotion in MDD.

### Brain Circuitry of Emotion Regulating Attentional Control

In this study, we explored the brain mechanisms underlying the attentional control influenced by emotion in dynamic attentional cognitive stages in MDD patients. The brain is known to be regulate this process and the underlying neural mechanism. According to the sLORETA technology, based on the dipole source location of ERP, we seek to find the related neural mechanisms in MDD.

Previous studies have suggested that human emotion management is under the control of ‘emotion brain’, including amygdala, ventral lateral prefrontal cortex (vlPFC), and its subgenual cingulates^21^. Substantial evidences presented amygdala as a foremost neural region, which is involved in detecting, processing, and maintaining the emotional information that is viewed as biological hallmark of the emotion brain. Further studies demonstrated that transient and clinical functions of subgenual cingulate and right prefrontal cortex were altered in negative mood as assessed by converging Positron Emission Tomography (PET)^51^. Wager also elaborated that ventral lateral PFC (vlPFC) was critical in the generation and regulation of emotion, especially, vlPFC was deemed to regulate the response to emotion through attentional-bias mechanism^15^. Recent studies reported that MDD patients exhibited impairments of affective regulation, which is involved in the frontal-limbic network^52,53^ and occipital-parietal regions during emotional face processing as revealed by fMRI analysis^53^.

Moreover, the cognitive control was involved in the brain regions of ‘cognitive brain’, including prefrontal system and anterior cingulate^54^, such as dorsolateral prefrontal cortex (DLPFC) and dorsal ACC^55^. The structural and metabolic changes in MDD patients occurred in the prefrontal brain regions, including the subgenual (SGPFC), medial (MPFC), and dorsolateral prefrontal cortex (DLPFC)^56^. The frontal-subcortical connectivity in depression is speculated to reflect an impaired cognitive regulation of mood^57^. The limbic-cortical reciprocity was responsible for mediating a top-down cognition. In addition, the oculomotor studies also indicated that prefrontal and cerebellar regions exhibited abnormalities in MDD^58^. The depressive individuals also showed impairment in lateral prefrontal cortex (LPFC) during a cognitive control of emotional information^7^. LPFC was involved in cognitive control during inhibition of competing responses or selected new information. According to the fMRI results, the right DLPFC hyperactivity was correlated with depressive severity while the left DLPFC was associated with negative emotional judgment. The imbalance of left-right DLPFC represented altered emotional-cognitive interaction in MDD^59^. Furthermore, the findings indicated that DLPFC played a major role in antidepressant treatment in depression during emotional processing, which suggested that the brain region was associated with cognitive control^14^. In addition, the caudal region of the ACC was associated with cognitive functions such as attention^60^. The limbic region activated in response to emotional information processing with neural imaging, while decreased DLPFC activity in response to cognitive tasks in unipolar major depressive disorder^61^.

The emotion-related brain and cognition-related brain are functionally related but structurally separate brain regions. However, they might interplay and modulate with each other in different processing stages. Various studies focused on the interaction in such duality between ‘cognitive brain’ and ‘emotional brain’. Previous studies demonstrated that the brain processed the information consecutively rather than sequentially^62^. Another recent study addressed that emotion and cognitive regulation were interwoven tightly^29^. Jin *et al*. provided similar results that cognitive and emotional processes partially shared common brain networks^62^. Thus, a dynamic integrated brain network could fulfill the demands of the cognitive tasks, thereby implying that the cognitive regulation of emotion was based on a closed circuitry in ‘emotion-cognition’ brain. These relative results led us to hypothesize that the emotion regulation cognitive control might be involved in a specific bidirectional regulative loop of brain circuitry, which begins from rostral temporal gyrus (RTG) to anterior ventral prefrontal cortex (AVPFC) in the brain of MDD patients during emotional face processing.

### Early attentional emotion processing-related brain regions

At an early attentional stage, the exogenous attention of MDD was biased towards the negative emotional stimuli. The salient emotional stimuli was under intensive focus in early information processing. This finding was in accordance with previous studies, which demonstrated such procedure as a ‘bottom-up’ stimuli-driven cognitive control^63^. Abundant evidences from fMRI studies revealed that the frontal prefrontal cortex, ACC, and amygdala were important emotion-related brain regions. Amygdala is a ‘biological marker’ of emotion processing, emotion regulation, and social behavior. Some groups showed amygdala as a core in processing emotional salient faces, especially fearful. They also provided direct evidence through Positron Emission Tomography (PET) that the neuron response in the left amygdala was greater over fearful than happy emotional faces as compared to the right side^64^. Further evidence showed that amygdala can modulate extrastriate cortex responses to fearful faces^15^. Our ERP results of N100 and P200 brain distribution and source locations might also reflect early perception of affective stimuli. As a result, the inferior temporal gyrus (ITG) and middle temporal gyrus (MTG) were activated during emotional expression processing. Among these regions, RTG might be the first key node of the ‘emotion-cognition’ circuitry in the brain of MDD patients.

### Late attentional control-related brain regions

At the late attentional stage, the MDD patients’ endogenous emotional regulation mechanism maintained the attentional control to sad emotional stimuli. In addition, after orienting the attention to emotional materials, the evoked internal mental state might give rise to coincident mood with external stimuli in MDD. Consequently, the sustained negative emotion might affect the cognitive control ability. Attentional control occurring in the late stage of processing is a type of cognitive control schema, which supports the endurance of emotional effects on individuals’ endogenous attention. Thus, such attentional control, termed as the top-down brain regulation strategies, might inhibit the attention disengaging from negative emotional stimuli to positive. The lateral prefrontal cortex (LPFC) is involved in this cognitive control of attention processing. Several groups reported that PFC is a vital brain region, serving as a modulator regulating the top-down procedure during the early visual processing, which effectuates the subsequent processing performance^65^. Scientists also demonstrated that the depressive symptoms are related to impaired lateral parietal PFC (LPPFC) during the cognitive control of emotional information^15^. The dorsolateral prefrontal cortex (DLPFC) is viewed as a core of cognition control and its activity may facilitate the effect of cognitive control therapy of MDD^66^. The abnormal activation in DLPFC was observed in depression during emotion processing tasks and cognitive control^21^. With our ERP and sLORETA results, a prolonged latency and higher amplitude of P300 provided a prominent evidence for inhibited cognitive abilities. The activated brain regions relative to P300 was localized in the dorsolateral parietal cortex (DLPFC). However, in MDD patients, anterior ventral prefrontal cortex (AVPFC) is involved in cognitive attentional control highly activated in MDD as compared to the HC group. Therefore, AVPFC might be another key intersection in ‘emotion-cognition’ brain circuitry.

In conclusion, the ‘emotion-cognition’ brain circuitry in attentional control cognitive processing of emotional faces in MDD patients might include two crucial nodes: RTG and AVPFC. This brain circuitry is responsible for mediating emotion and attention. According to different processing stages, this circuitry alters dynamically. In the early attentional stage, emotion node (RTG) was activated. Subsequently, in the late attentional state, cognition node (AVPFC) was activated for energy. These two parts might interplay with each other dynamically. This bidirectional regulation loop of brain circuitry might contribute to reveal the early and late potential mechanism underlying the brain neurocognition of attention in MDD patients.

Nevertheless, the present study has some limitations that should be resolved in future research. Firstly, different methods of data acquisition might be used. Although ERP has high temporal resolution in during the neural response in relation to emotional regulation; the low spatial resolution renders difficulty in describing accurate neural circuitry. Therefore, we cannot determine the accurate ‘emotion-cognition’ brain neural circuitry from these ERP results. Sufficient results obtained based on the ERP topographies indicates only rough brain regions. In order to overcome this limitation and obtain activated neural circuitry rigidly, we used the sLORETA to identify the brain functional regions of correlative emotion-cognition control. In future studies, it might use precise spatial-temporal method based on fMRI combined with ERP. Secondly, in addition to the dot-probe paradigm, other psychological attention paradigms can also be performed to identify the relative ERP component in relation to emotion regulation. Thirdly, to gain a deep insight on cognition of depression, more subtypes of depressive patients should be included. Despite the above shortcomings, this preliminary study, which utilized the ERP dot-probe paradigm investigating the correlation between emotion and attention in MDD can clearly indicate some potential neurocognitive mechanisms.

Taken together, the present study demonstrated that emotion regulates attentional control in the early and late stages of cognitive processing in MDD patients. Thus, future studies should investigate the specific forms of attentional control as well as brain mechanisms of emotion-cognitive interactive modulation.

## Conclusion

Recently, emotion and cognition has been under intensive focus with respect to cognitive neuroscience. Notable work have been carried out in this area, especially MDD patients. In this study, the amplitude and peak latency of ERP components including N100, P200, and P300 are modulated by emotional stimulus. Consequently, we indicated that emotion might regulate the attentional control in the early and late stages of cognitive processing. Additionally, an ‘emotion-cognition’ brain circuitry activated in RTG-AVPFC is a potential brain mechanism responsible for emotion regulation in MDD. This circuitry based on interactions between activated regional brains might contribute to emotion-cognition regulation in MDD. From the perspective of neuroscience theory, this circuitry is a closed bidirectional neural loop responsible for regulating emotion and attentional control ability in MDD.

These ERP component-related findings have important clinical treatment implications, which can be used as diagnostic parameters to assess the cognitive function in MDD patients clinically. Determining whether the potential brain regulation circuitry contributes to the early warning in high-risk depressive individuals as well as improves psychological intervention effects of cognitive therapy in MDD is yet to be elucidated.

## Electronic supplementary material

Supplementary Information
