# Supplementary material for: Emotion Regulating Attentional Control Abnormalities In Major Depressive Disorder: An Event-Related Potential Study
Source: Sci Rep. 2017 Oct 19;7:13530. doi: 10.1038/s41598-017-13626-3 (PMC5648876; doi:10.1038/s41598-017-13626-3)
Supplement: Supplementary file 1 — Supplementary Information [file 41598_2017_13626_MOESM1_ESM.doc]

**Supplementary Information**

**Emotion regulating attentional control abnormalities in Major Depressive Disorder: An Event-Related Potential study**

Bin Hu, Juan Rao, Xiaowei Li, Tong Cao, Jianxiu Li, Dennis Majoe, Jürg Gutknecht

**Supplementary Figure 1**

**Brain regions associated with N100 component from sLORETA images compared with MDD (left panel) and HC (right panel) at 100 ms.** Results presented as six pictures from six different spacial positions (top, bottom, front, back, left and right) which were used for outputs under three emotional facial compound stimuli modules (Happy-Neutral, Fear-Neutral and Sad-Neutral) modules. Coordinates in MNI space was in mm. Corrected p<0.05.

Figure 6A showed six spacial positions (top, bottom, front, back, left and right) of brain images from all six-picture outputs in Supplementary Figure1.

**
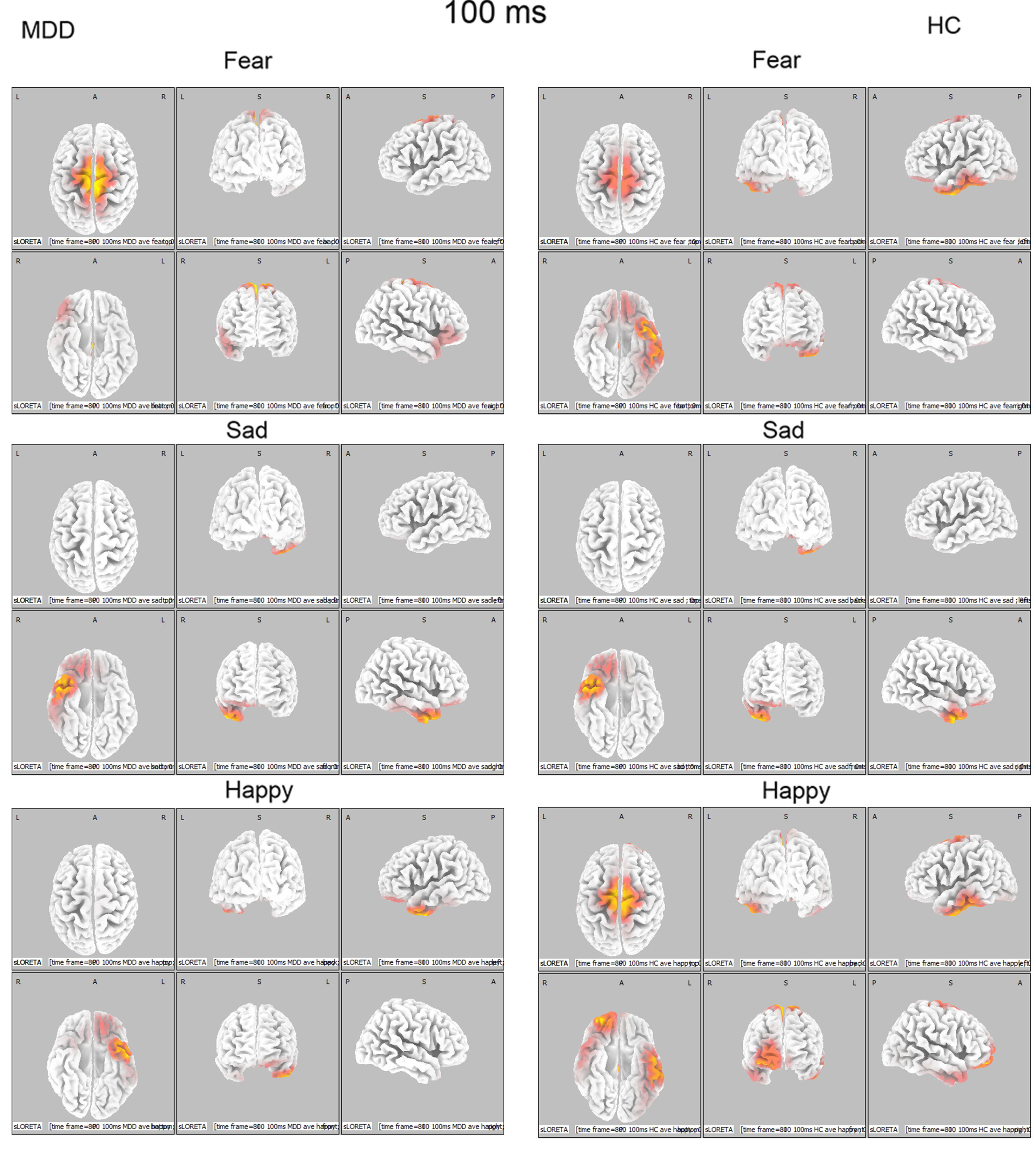
**

**Supplementary Figure 2**

**Brain regions associated with P200 component from sLORETA images compared with MDD (left panel) and HC (right panel) at 200 ms.** Results presented six pictures from six different spacial positions (top, bottom, back, front, left and right) which were used for outputs under three emotional facial compound stimuli modules (Happy-Neutral, Fear-Neutral and Sad-Neutral) modules. Coordinates in MNI space was in mm. Corrected p<0.05.

Figure 7A showed six spacial positions (top, bottom, front, back, left and right) of brain images from all six-picture outputs in Supplementary Figure 2.

**
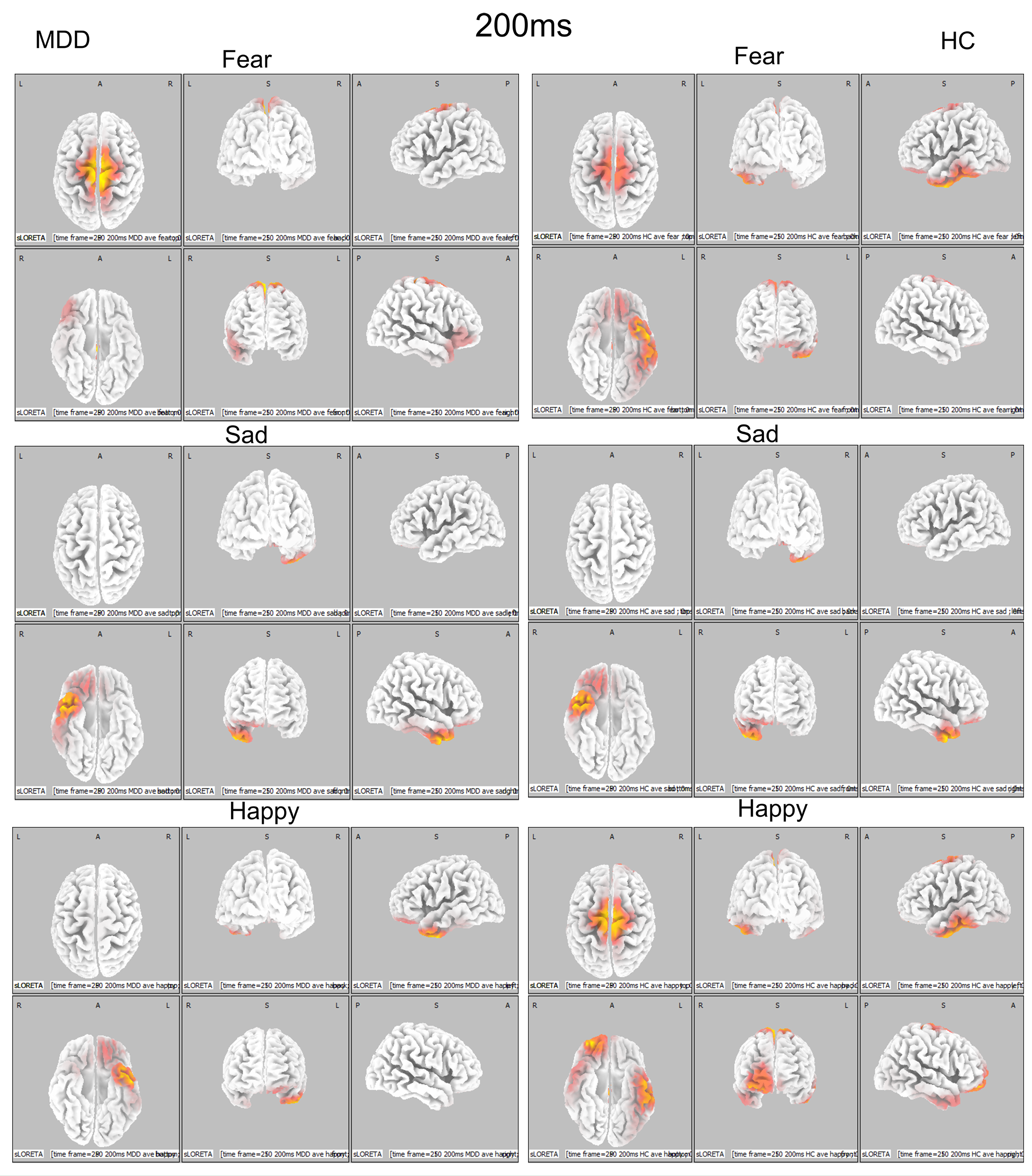
**

**Supplementary Figure 3a**

**Brain regions associated with P300 component in from sLORETA images compared with MDD (left panel) and HC (right panel) at 300 ms under Emotion-Congruent condition.** Results presented six pictures from six different spacial positions (top, bottom, back, front, left and right) which were used for outputs under three emotional facial compound stimuli modules (Happy-Neutral, Fear-Neutral and Sad-Neutral) modules. Coordinates in MNI space was in mm. Corrected p <0.05

Figure 8A showed six basic spacial positions (top, bottom, front, back, left and right) of brain images from all six-picture outputs in Supplementary Figure 3a.

**
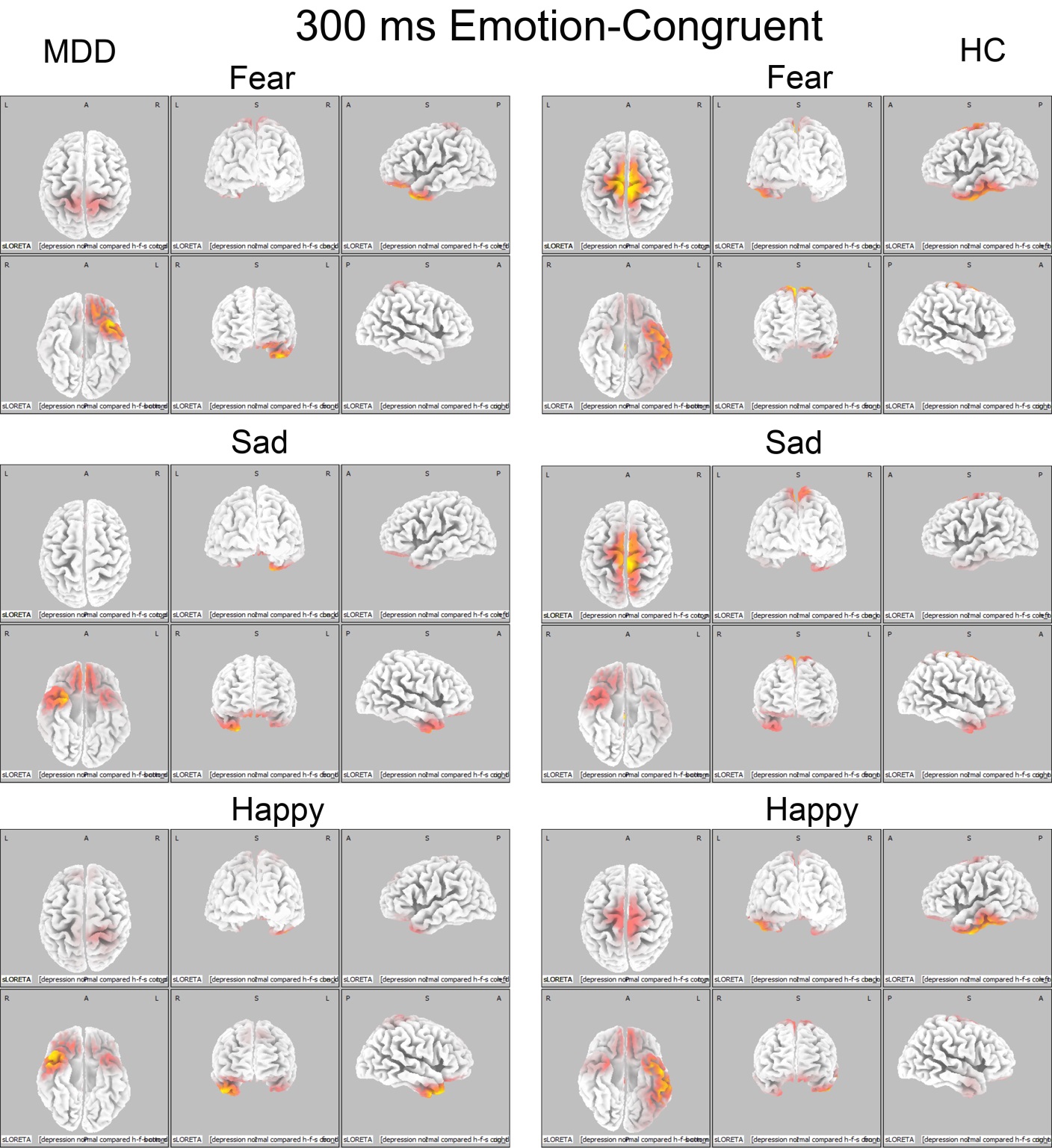
**

**Supplementary Figure 3b**

**Brain regions associated with P300 component from sLORETA images compared with MDD (left panel) and HC (right panel) at 300 ms under Emotion-Incongruent condition.** Results presented six pictures from six different space positions (top, bottom, back, front, left and right) which were used for outputs under three emotional facial compound stimuli modules (Happy-Neutral, Fear-Neutral and Sad-Neutral) modules. Coordinates in MNI space was in mm. Corrected p <0.05

Figure 8B showed six basic spacial positions (top,bottom, front, back, left and right) of brain images from all six-picture outputs in Supplementary Figure 3b.


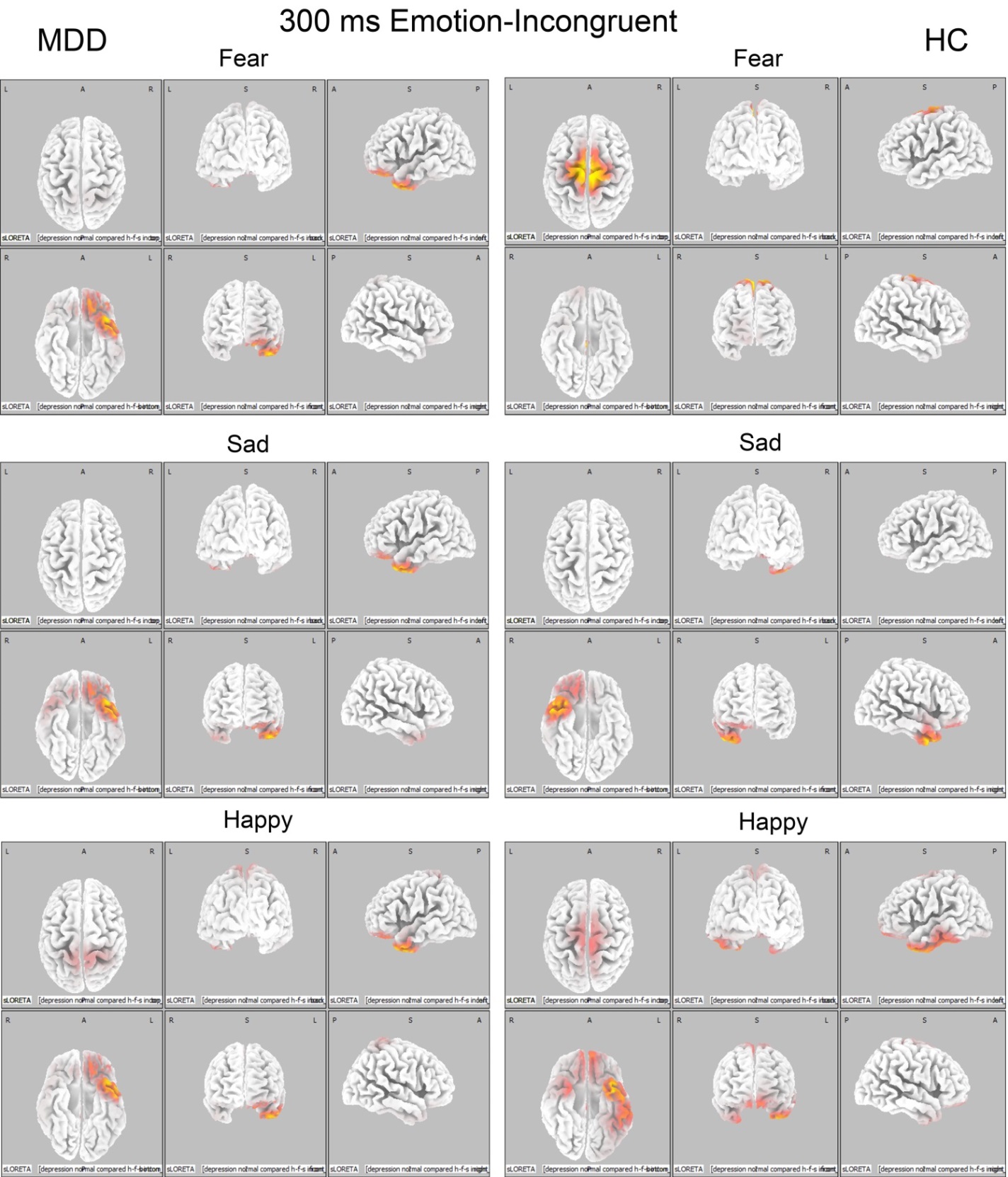


**Supplementary Figure 4**

**Brain regions activated by Sad emotion modules from 200 ms to 300 ms shown through sLORETA image compared with MDD (top panel) and HC (bottom panel) under Emotion-Congruent condition.** Results presented six pictures from six different spacial positions (top, bottom, back, front, left and right) which were used for outputs under three emotional facial compound stimuli modules (Happy-Neutral, Fear-Neutral and Sad-Neutral) modules. Coordinates in MNI space was in mm. Corrected p <0.05

Figure 10 presented four basic spacial positions (top, bottom, front and right) of brain images from all six-picture outputs in Supplementary Figure 4.


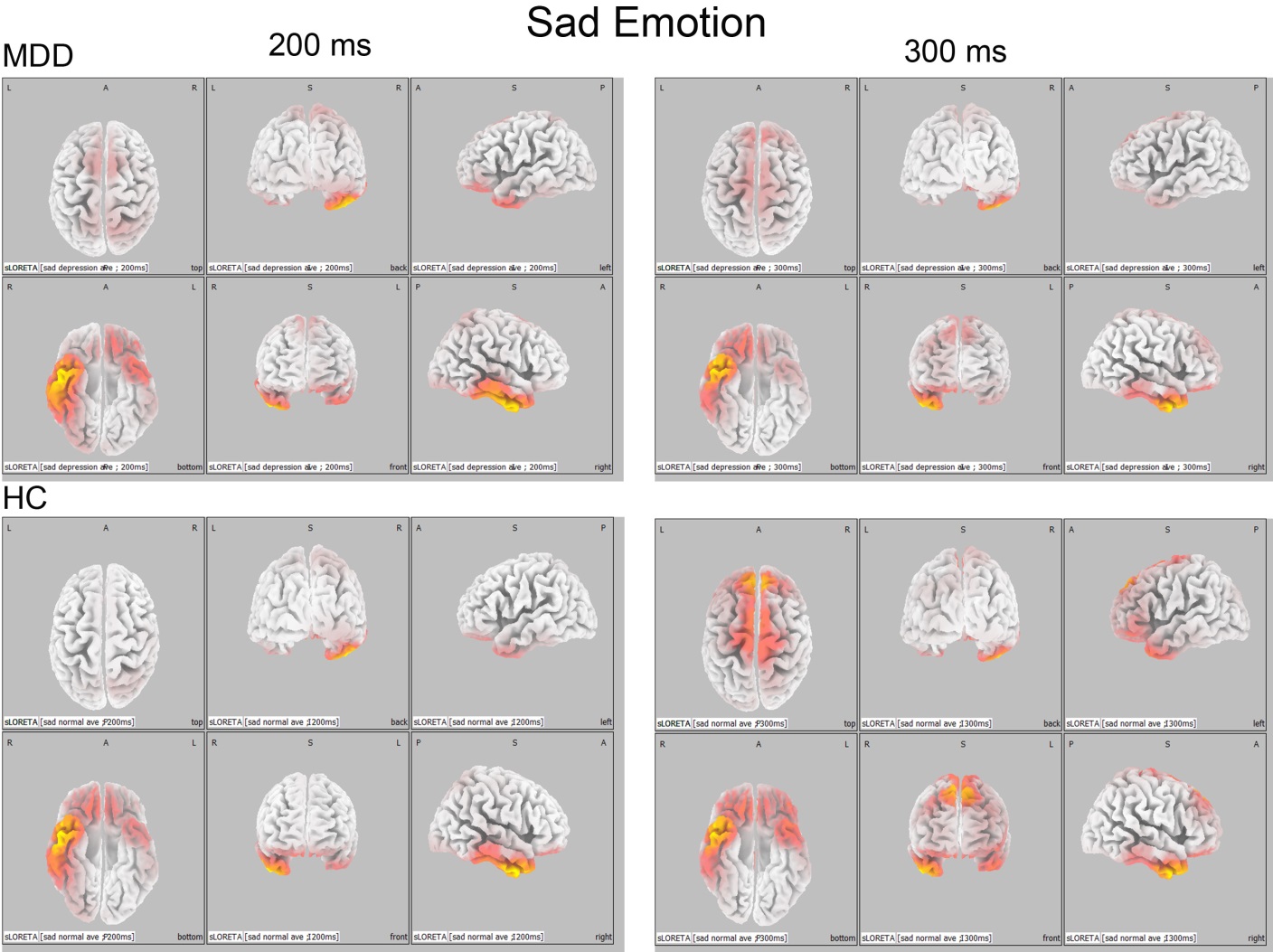


**Supplementary Table 1**

**Main effects and interactions on amplitude and latency of N100 and P200 analyzed by ANOVA.** The results were reported and discussed in the Result section of the manuscripts.

**a. Main effects**

|  | Factor | | Multivariate Tests | | Between-Subjects Effects | | | | | | |
| --- | --- | --- | --- | --- | --- | --- | --- | --- | --- | --- | --- |
|  |  |  | Sig. | | Amplitude | | | Latency | | | |
|  |  |  |  |  | df | F | Sig. | df | F | Sig. | |
| N100 | Group |  | .038 | | 1 | 5.503 | .019 | 1 | .244 | .621 | |
|  | Emotion |  | .001 | | 2 | 7.018 | .001 | 2 | .779 | .459 | |
|  | ROI |  | .000 | | 3 | 13.858 | .000 | 3 | 11.023 | .000 | |
|  |  |  |  | |  |  |  |  |  |  | |
|  | Group * Emotion | | .145 | | 2 | .394 | .675 | 2 | 2.888 | .056 | |
|  | Group * ROI | | .030 | | 3 | .704 | .550 | 3 | 5.616 | .001 | |
|  | Emotion * ROI | | .995 | | 6 | .118 | .994 | 6 | .375 | .895 | |
|  | Group * Emotion * ROI | | | .999 | 6 | .141 | .991 | 6 | .175 | | .983 |
|  |  |  |  | |  |  |  |  |  |  | |
| P200 | Group |  | .000 | | 1 | 50.775 | .000 | 1 | .479 | .489 | |
|  | Emotion |  | .018 | | 2 | 5.133 | .006 | 2 | .791 | .454 | |
|  | ROI |  | .000 | | 3 | 8.001 | .000 | 3 | 3.171 | .024 | |
|  |  |  |  | |  |  |  |  |  |  | |
|  | Group * Emotion | | .131 | | 2 | 1.914 | .148 | 2 | 1.326 | .266 | |
|  | Group * ROI | | .109 | | 3 | 3.442 | .016 | 3 | .145 | .933 | |
|  | Emotion * ROI | | .997 | | 6 | .092 | .997 | 6 | .315 | .930 | |
|  | Group * Emotion * ROI | | | .993 | 6 | .213 | .973 | 6 | .281 | .946 | |
| a. Design: Intercept + Group + Emotion + ROI + Group * Emotion + Group * ROI + Emotion * ROI + Group * Emotion * ROI | | | | | | | | | | | |
| b. The statistic is an upper bound on F that yields a lower bound on the significance level. | | | | | | | | | | | |
| c.Corrected model of N100 amplitude is Type III Sum of Squares, R Squared = .076 (Adjusted R Squared = .051), N100 latency is Type III Sum of Squares, R Squared = .068 (Adjusted R Squared = .042) | | | | | | | | | | | |
| d. Corrected model of P200 amplitude is Type III Sum of Squares, R Squared = .110 (Adjusted R Squared = .086), P200 latency is Type III Sum of Squares, R Squared = .022 (Adjusted R Squared = -.005) | | | | | | | | | | | |

**b. Interaction Effects**

| Factor | Levels of Factor | | Pairwise Comparisons | | | | | | | |
| --- | --- | --- | --- | --- | --- | --- | --- | --- | --- | --- |
|  |  |  | N100 | | | | P200 | | | |
|  |  |  | Amplitude | | Latency | | Amplitude | | Latency | |
|  |  |  | Std.Error | Sig. | Std.Error | Sig. | Std.Error | Sig. | Std.Error | Sig. |
| Group | MDD | HC | .199 | .019 | .915 | .621 | .258 | .000 | 1.045 | .489 |
|  | HC | MDD | .199 | .019 | .915 | .621 | .258 | .000 | 1.045 | .489 |
| Emotion | Fear | Happy | .243 | .144 | 1.120 | .692 | .316 | .265 | 1.280 | .220 |
|  |  | Sad | .243 | .000 | 1.120 | .222 | .316 | .002 | 1.280 | .709 |
|  | Happy | Fear | .243 | .144 | 1.120 | .692 | .316 | .265 | 1.280 | .220 |
|  |  | Sad | .243 | .024 | 1.120 | .409 | .316 | .041 | 1.280 | .394 |
|  | Sad | Fear | .243 | .000 | 1.120 | .222 | .316 | .002 | 1.280 | .709 |
|  |  | Happy | .243 | .024 | 1.120 | .409 | .316 | .041 | 1.280 | .394 |
| ROI | Central | Frontal | .243 | .001 | 1.120 | .066 | .316 | .934 | 1.280 | .225 |
|  |  | Parietal | .281 | .052 | 1.293 | .000 | .364 | .000 | 1.478 | .062 |
|  |  | Prefrontal | .281 | .000 | 1.293 | .348 | .364 | .083 | 1.478 | .404 |
|  | Frontal | Central | .243 | .001 | 1.120 | .066 | .316 | .934 | 1.280 | .225 |
|  |  | Parietal | .281 | .000 | 1.293 | .000 | .364 | .000 | 1.478 | .004 |
|  |  | Prefrontal | .281 | .276 | 1.293 | .514 | .364 | .072 | 1.478 | .828 |
|  | Parietal | Central | .281 | .052 | 1.293 | .000 | .364 | .000 | 1.478 | .062 |
|  |  | Frontal | .281 | .000 | 1.293 | .000 | .364 | .000 | 1.478 | .004 |
|  |  | Prefrontal | .314 | .000 | 1.446 | .000 | .407 | .021 | 1.653 | .016 |
|  | Prefrontal | Central | .281 | .000 | 1.293 | .348 | .364 | .083 | 1.478 | .404 |
|  |  | Frontal | .281 | .276 | 1.293 | .514 | .364 | .072 | 1.478 | .828 |
|  |  | Parietal | .314 | .000 | 1.446 | .000 | .407 | .021 | 1.653 | .016 |
| Based on estimated marginal means | | | | | | | | | | |
| *. The mean difference is significant at the .05 level. | | | | | | | | | | |
| a. Adjustment for multiple comparisons: Least Significant Difference (equivalent to no adjustments). | | | | | | | | | | |

**Supplementary Table 2**

**Main effects and interaction on amplitude and latency of P300 analyzed by ANOVA.** The results were reported and discussed in the Result section of the manuscripts.

**a. Main effects**

| P300 | Factor | Between-Subjects Effects | | | | | |
| --- | --- | --- | --- | --- | --- | --- | --- |
|  |  | Amplitude | | | Latency | | |
|  |  | df | F | Sig. | df | F | Sig. |
|  | Emotion-Congruent Condition |  |  |  |  |  |  |
|  | Group | 1 | .461 | .498 | 1 | 2.352 | .126 |
|  | ROI | 2 | 3.148 | .044 | 2 | 4.717 | .009 |
|  | Emotion-Congruent | 2 | 6.056 | .003 | 2 | .703 | .496 |
|  |  |  |  |  |  |  |  |
|  | Group * ROI | 2 | 1.203 | .301 | 2 | 4.504 | .012 |
|  | Group * Emotion-Congruent | 2 | 11.340 | .000 | 2 | 4.511 | .011 |
|  | ROI * Emotion-Congruent | 4 | .100 | .982 | 4 | .720 | .578 |
|  | Group * ROI * Emotion-Congruent | 4 | .451 | .772 | 4 | .251 | .909 |
|  | Emotion-Incongruent Condition |  |  |  |  |  |  |
|  | Group | 1 | .187 | .665 | 1 | 9.978 | .002 |
|  | ROI | 2 | 1.799 | .167 | 2 | 6.040 | .003 |
|  | Emotion-Incongruent | 2 | 1.560 | .211 | 2 | .409 | .665 |
|  |  |  |  |  |  |  |  |
|  | Group * ROI | 2 | 2.462 | .086 | 2 | .800 | .450 |
|  | Group * Emotion-Incongruent | 2 | 3.654 | .027 | 2 | 3.269 | .039 |
|  | ROI * Emotion-Incongruent | 4 | .040 | .997 | 4 | .326 | .861 |
|  | Group * ROI * Emotion-Incongruent | 4 | .217 | .929 | 4 | .136 | .969 |
| a. Emotion-Congruent Condition: P300 amplitude of Corrected model is Type III Sum of Squares, R Squared = .086 (Adjusted R Squared = .056), P300 latency of Corrected model is Type III Sum of Squares, R Squared = .066 (Adjusted R Squared = .035) | | | | | | | |
| b. Emotion-Incongruent Condition: P300 amplitude of Corrected model is Type III Sum of Squares, R Squared = .041 (Adjusted R Squared = .010), P300 latency of Corrected model is Type III Sum of Squares, R Squared = .062 (Adjusted R Squared = .030) | | | | | | | |

**b. Interaction Effects**

| Factor | Levels of Factor | | Pairwise Comparisons | | | |
| --- | --- | --- | --- | --- | --- | --- |
|  |  |  | Amplitude | | Latency | |
|  |  |  | Std.Error | Sig. | Std.Error | Sig. |
| Emotion-Congruent |  |  |  |  |  |  |
| Group | MDD | HC | .335 | .498 | 2.463 | .126 |
|  | HC | MDD | .335 | .498 | 2.463 | .126 |
| Emotion | Fear_Cong | Happy_Cong | .410 | .317 | 3.017 | .244 |
|  |  | Sad_Cong | .410 | .017 | 3.017 | .692 |
|  | Happy_Cong | Fear_Cong | .410 | .317 | 3.017 | .244 |
|  |  | Sad_Cong | .410 | .001 | 3.017 | .442 |
|  | Sad_Cong | Fear_Cong | .410 | .017 | 3.017 | .692 |
|  |  | Happy_Cong | .410 | .001 | 3.017 | .442 |
| ROI | Central | Occipital | .441 | .033 | 3.240 | .003 |
|  |  | Parietal | .394 | .023 | 2.898 | .193 |
|  | Occipital | Central | .441 | .033 | 3.240 | .003 |
|  |  | Parietal | .394 | .921 | 2.898 | .037 |
|  | Parietal | Central | .394 | .023 | 2.898 | .193 |
|  |  | Occipital | .394 | .921 | 2.898 | .037 |
| Emotion_Incongruent |  |  |  |  |  |  |
| Group | MDD | HC | .297 | .665 | 2.598 | .002 |
|  | HC | MDD | .297 | .665 | 2.598 | .002 |
| Emotion | Fear_Incong | Happy_Incong | .353 | .063 | 3.092 | .649 |
|  |  | Sad_Incong | .353 | .205 | 3.092 | .686 |
|  | Happy_Incong | Fear_Incong | .353 | .063 | 3.092 | .649 |
|  |  | Sad_Incong | .353 | .551 | 3.092 | .390 |
|  | Sad_Incong | Fear_Incong | .353 | .205 | 3.092 | .686 |
|  |  | Happy_Incong | .353 | .551 | 3.092 | .390 |
| ROI | Central | Occipital | .391 | .063 | 3.418 | .001 |
|  |  | Parietal | .349 | .179 | 3.057 | .009 |
|  | Occipital | Central | .391 | .063 | 3.418 | .001 |
|  |  | Parietal | .349 | .464 | 3.057 | .248 |
|  | Parietal | Central | .349 | .179 | 3.057 | .009 |
|  |  | Occipital | .349 | .464 | 3.057 | .248 |
| *. The mean difference is significant at the .05 level.  a. Adjustment for multiple comparisons: Least Significant Difference (equivalent to no adjustments) | | | | | | |

**Supplementary Table 3**

**Brain regions which showed significant differences in activation between MDD and HC group at N100 and P200 latency. The results were reported and discussed in the Result section of the manuscripts. This table was cited as Table 2 in the manuscript.**

| Condition | N1 | | | | P2 | | | |
| --- | --- | --- | --- | --- | --- | --- | --- | --- |
|  | Lobe | Anatomical Region | BA | MNI  Coordinates | Lobe | Anatomical Region | BA | MNI  Coordinates |
| Emotion (MDD<HC) | |  |  |  |  |  |  |  |
| Fear | Frontal | IFG | 45 | (40,20,5) | Frontal | PcG | 6 | (40,-10,45) |
|  |  |  | 47 | (40,22,4) |  |  | 2 | (40,-19,48) |
|  | Temporal | STG | 38 | (14,40,-37) |  | MFG | 6 | (38,0,43) |
|  |  | MTG | 21 | (40,2,-35) |  |  |  |  |
|  |  | ITG | 20 | (40,-4,-41) |  |  |  |  |
| Sad | Occipital | MOG | 18 | (-15,-90,15) | Parietal | PCG | 1 | (50,-25,60) |
|  | Paracentral | PcL | 5 | (-2,-34,57) |  |  |  |  |
|  |  |  | 6 | (-2,-24,65) |  |  |  |  |
|  | Frontal | MFG | 6 | (1,-24,56) |  |  |  |  |
|  |  |  | 11 | (-35,41,-21) |  |  |  |  |
|  | Temporal | STG | 38 | (-35,19,-33) |  |  |  |  |
| Happy | Limbic | Pc | 31 | (-15,-60,30) |  |  |  |  |
|  | Occipital | Pc | 31 | (-13,-92,16) | Occipital | Cuneus | 19 | (-15,-90,25) |
|  |  | MOG | 18 | (40,-10,45) |  |  | 18 | (-15,-82,26) |

IFG: Inferior Frontal Gyrus; STG: Superior Temporal Gyrus; MTG: Middle Temporal Gyrus; ITG: Inferior Temporal Gyrus; MOG: Middle Occipital Gyrus; PcL: Paracentral Lobule; MFG: Middle Frontal Gyrus; STG: Superior Temporal Gyrus; BA: Brodmann Area; MNI: Montreal Neurological Institute coordinates; Corrected p<0.05

**Supplementary Table 4**

**Brain regions which showed significant differences in activation between MDD and HC group at P300 latency under Emotion-Congruent condition. The results were reported and discussed in the Result section of the manuscripts. This table was cited as Table 3 in the manuscript.**

| Condition | Lobe | Anatomical Region | BA | MNI Coordinates |
| --- | --- | --- | --- | --- |
| Emotion-Congruent (MDD>HC) | |  |  |  |
| Fear-Con | Parietal | SPL | 7 | (-30,-80,45) |
|  |  | Pc | 7 | ( 8,-68,39) |
|  |  | IPL | 7 | (-41,-68,74) |
|  |  |  | 40 | (-45,-66,37) |
|  | Temporal | STG | 39 | (-45,-61,29) |
|  | Occipital | STG | 19 | (-41,-78,23) |
| Sad-Con | Occipital | MOG | 19 | (45,-85,5) |
|  |  | Cuneus | 17 | (37,-43,54) |
|  | Parietal | IPL | 40 | (12,-92,7) |
| Happy-Con | Frontal | IFG | 9 | (50,5,35) |
|  |  | MFG | 9 | (50,6,39) |
|  |  | PcG | 6 | (50,1,33) |

SPL: Superior Parietal Lobule; Pc: Precuneus; IPL: Inferior Parietal Lobule;

STG: Superior Temporal Gyrus; MOG: Middle Occipital Gyrus; IFG: Inferior Frontal Gyrus; MFG: Middle Frontal Gyrus; MTG: Middle Temporal Gyrus; ITG: Inferior Temporal Gyrus;; PcL: Paracentral Lobule; PcG: Postcentral Gyrus; BA: Brodmann Area; MNI: Montreal Neurological Institute coordinates; Corrected p<0.05

**Supplementary Table 5**

**Main effects and interaction on Reaction Time (RT) between MDD and HC**

**under Emotion-Congruent and Emotion-Incongruent conditions.** The results were reported and discussed in the Result section of the manuscripts. This table was cited as Table 1 in the manuscript.

| Factor | Levels of Factor | | Post Hoc Tests  (LSD) | | Pairwise Comparisons | | |
| --- | --- | --- | --- | --- | --- | --- | --- |
|  |  |  | Std.Error | Sig. | Std.Error | | Sig. |
| Emotion-Congruent | |  |  |  |  | |  |
| Group | MDD | HC | 5.042 | .000 | 2.911 | .000 | |
|  | HC | MDD | 5.042 | .000 | 2.911 | .000 | |
| Emotion | Fear_Cong | Happy_Cong | 3.493 | .562 | 3.565 | .470 | |
|  |  | Sad_Cong | 3.493 | .096 | 3.565 | .239 | |
|  | Happy_Cong | Fear_Cong | 3.493 | .562 | 3.565 | .470 | |
|  |  | Sad_Cong | 3.493 | .025 | 3.565 | .058 | |
|  | Sad_Cong | Fear_Cong | 3.493 | .096 | 3.565 | .239 | |
|  |  | Happy_Cong | 3.493 | .025 | 3.565 | .058 | |
| Emotion-Incongruent | |  |  |  |  |  | |
| Group | MDD | HC | 7.669 | .012 | 4.427 | .000 | |
|  | HC | MDD | 7.699 | .012 | 4.427 | .000 | |
| Emotion | Fear_Incong | Happy_Incong | 5.312 | .544 | 5.421 | .655 | |
|  |  | Sad_Incong | 5.313 | .037 | 5.422 | .126 | |
|  | Happy_Incong | Fear_Incong | 5.312 | .544 | 5.421 | .655 | |
|  |  | Sad_Incong | 5.313 | .140 | 5.422 | .279 | |
|  | Sad_Incong | Fear_Incong | 5.313 | .037 | 5.422 | .126 | |
|  |  | Happy_Incong | 5.313 | .140 | 5.422 | .279 | |
| *. The mean difference is significant at the .05 level. | | | | | | | |
| Adjustment for multiple comparisons: Least Significant Difference (equivalent to no adjustments). | | | | | | | |
